# Birds Generally Carry a Small Repertoire of Bitter Taste Receptor Genes

**DOI:** 10.1093/gbe/evv180

**Published:** 2015-09-04

**Authors:** Kai Wang, Huabin Zhao

**Affiliations:** Department of Ecology, College of Life Sciences, Wuhan University, Wuhan, China

**Keywords:** bitter taste, *Tas2r*, diet, birds, feeding ecology

## Abstract

As they belong to the most species-rich class of tetrapod vertebrates, birds have long been believed to possess an inferior taste system. However, the bitter taste is fundamental in birds to recognize dietary toxins (which are typically bitter) in potential food sources. To characterize the evolution of avian bitter taste receptor genes (*Tas2r*s) and to test whether dietary toxins have shaped the repertoire size of avian *Tas2r*s, we examined 48 genomes representing all but 3 avian orders. The total number of *Tas2r* genes was found to range from 1 in the domestic pigeon to 12 in the bar-tailed trogon, with an average of 4, which suggested that a much smaller *Tas2r* gene repertoire exists in birds than in other vertebrates. Furthermore, we uncovered a positive correlation between the number of putatively functional *Tas2r*s and the abundance of potential toxins in avian diets. Because plant products contain more toxins than animal tissues and insects release poisonous defensive secretions, we hypothesized that herbivorous and insectivorous birds may demand more functional *Tas2r*s than carnivorous birds feeding on noninsect animals. Our analyses appear to support this hypothesis and highlight the critical role of taste perception in birds.

## Introduction

Sensing the external environment is of critical importance for the survival of animals. The five traditional senses in vertebrates of taste, sight, smell, sound, and touch recognize environmental cues that trigger or adjust animal behaviors accordingly. The sense of taste is specialized to evaluate the chemical components in potential food resources, which evoke appetitive or aversive reactions to ensure the ingestion of nutrients rather than poisonous substances (Yarmolinsky et al. 2009). The five basic taste modalities in vertebrates are bitter, sweet, umami, sour, and salty (Lindemann 1996; Bachmanov and Beauchamp 2007). Of them, bitter taste is dedicated to identifying bitter-tasting chemicals, such as plant alkaloids and insect defensive secretions (Garica and Hankins 1975; de Jong et al. 1991; Glendinning 1994), which are potentially poisonous to animals. Thus, bitter taste is a critical natural defense preventing the ingestion of toxic or harmful substances, which are typically bitter in nature (Garica and Hankins 1975; Glendinning 1994).

Bitter taste is conferred by the physical interaction of bitter chemicals with a group of G protein-coupled receptors (Tas2rs) that are encoded by members of the type 2 taste receptor genes (*Tas2r*s) (Adler et al. 2000; Chandrashekar et al. 2000; Matsunami et al. 2000). It is generally believed that the repertoire size of taste receptors is intimately associated with the external environment that animals inhabit. Indeed, the total number of *Tas2r*s, varying substantially from 3 in the chicken to 69 in the guinea pig, and the number of putatively functional *Tas2r*s, ranging from 0 in the dolphin to 51 in the frog, are positively correlated with the amount of plant materials in diets across vertebrates (Li and Zhang 2014). In addition, frequent expansions of *Tas2r*s in some primate lineages were also assumed to link with the development of plant feeding (Hayakawa et al. 2014). These findings agreed with the assumption that plant materials contain more bitter compounds than animal tissues (Glendinning 1994; Wang et al. 2004) and supported the hypothesis that bitter tastants have driven the evolution of the *Tas2r* gene repertoire in vertebrate animals (Li and Zhang 2014). Thus, bitter taste is a good model to evaluate how the chemosensory receptor gene repertoire was shaped by dietary or environmental factors. However, within vertebrates, birds were reported to possess a smaller *Tas2r* gene repertoire compared with mammals (Go 2006; Li and Zhang 2014; Zhao et al. 2015). Specifically, the members of *Tas2r*s have been examined thus far in 16 birds with fully sequenced genomes, ranging from 1 in the dove (i.e., domestic pigeon) to 12 in the hummingbird (Zhao et al. 2015), with an average of 5, whereas the *Tas2r*s of mammals vary in number from 10 in the dolphin (or platypus) to as many as 69 in the guinea pig, with an average of 31 (Li and Zhang 2014). Similarly, the number of putatively functional *Tas2r*s in birds (a mean of 3.9 and a range of 0–10) is generally lower than that in mammals (a mean of 19.5 and a range of 0–36) (Li and Zhang 2014; Zhao et al. 2015). The taste receptor genes in birds have not been well characterized thus far because the avian taste system has long been believed to be largely reduced, as inferred from a low number of taste buds and the absence of teeth (Roura et al. 2013). Indeed, chickens show an indifference to sweet stimuli in behavioral tests (Ganchrow et al. 1990), and the gene encoding the sweet taste receptor is missing from its genome (Shi and Zhang 2006). Intriguingly, some birds, such as the white-throated sparrow (*Zonotrichia albicollis*), were found to have 18 putatively functional *Tas2r*s, a number that is comparable with many mammals (Davis et al. 2010). Such a dramatic change in the *Tas2r* repertoire size may not be uncommon in birds with the increasing number of additional avian genomes being deciphered. To characterize the origin and evolution of avian *Tas2r*s and to test whether dietary toxins have shaped the repertoire size of avian *Tas2r*s, we examined 48 avian genomes, representing nearly all avian orders (Jarvis et al. 2014; Zhang et al. 2014), to understand the evolution of *Tas2r*s in birds. We found that the putatively functional *Tas2r* repertoire size in birds is positively correlated with the abundance of potential toxins in their diets, although birds generally carry a small number of *Tas2r*s.

## Materials and Methods

### Diet Classification

Very few bird species feed on a single type of food; the composition of avian diets is significantly influenced by food availability, seasonal changes, age, and other factors (DeGolier et al. 1999). To be consistent with earlier studies, we followed the method of Wilson (1974), which is based on the stomach contents: When a food type predominated in the stomachs of 51% or more of samples, the bird species was assigned to that food category (DeGolier et al. 1999). We did our best to search the quantitative data regarding diet composition in the literature (supplementary table S1, Supplementary Material online) and the Animal Diversity Web (http://animaldiversity.org, last accessed June 30, 2015); when such data were not available but a food type was the most abundant component in the description of food habits, we assigned the species to that food category. Because plant products may contain more toxins than animal tissues and insects release poisonous defensive secretions, we did not differentiate qualitatively among birds that feed on different plant products, whereas birds that eat animals were divided into insectivores (insect eaters) and carnivores (noninsect animal eaters). As a result, we classified birds into seven categories according to their food habits (supplementary table S1, Supplementary Material online): 1) Folivores (referring to bird species that mostly eat leaves); 2) frugivores (referring to those birds that mostly feed on fruits); 3) granivores (referring to birds that predominantly eat the seeds of plants); 4) nectarivores (referring to those that mainly feed on the sugar-rich nectar); 5) insectivores (referring to any bird species that predominantly feeds on insects); 6) carnivores (referring to any bird species that predominantly eats noninsect animals, such as piscivores); and 7) omnivores (referring to any bird species that eats insects, noninsect animals, and plant products without quantitative records).

### Genome Data

A total of 48 avian genome sequences, including 45 that were recently released (Jarvis et al. 2014; Zhang et al. 2014) and 3 that were published earlier (Hillier et al. 2004; Dalloul et al. 2010; Warren et al. 2010), were retrieved from the Avian Phylogenomics Project (http://avian.genomics.cn/en/, last accessed January 30, 2015). The three genome sequences of crocodilians, representing the closest outgroup of all extant birds (Green et al. 2014), were obtained from the National Center for Biotechnology Information database with the following accession numbers: AKHW00000000 (*Alligator mississippiensis*), JRXG00000000 (*Crocodylus porosus*), and JRWT00000000 (*Gavialis gangeticus*).

### Gene Identification

Vertebrate *Tas2r*s are single-exon genes that encode bitter taste receptors characterized by seven transmembrane domains (Adler et al. 2000; Chandrashekar et al. 2000; Matsunami et al. 2000). To identify the *Tas2r* repertoire in each of the 48 birds and in 3 outgroup species of crocodilians, we followed an earlier study (Shi and Zhang 2006) with minor modifications. First, we used full-length Tas2r protein sequences from human, mouse, zebra finch, chicken, lizard, frog, and zebra fish as queries to conduct TBLASTN searches against each of the 51 genomes, with a cutoff *e*-value of 1 × 10^−^^10^. Second, we filtered the redundant sequences that hit on the same genomic regions and discarded the blast hits that were shorter than 200 nt. Third, the remaining blast hits were extracted from the genomes and extended in both 5′ and 3′ directions. Those with more than 270 codons and a putative start and stop codon are intact genes; those with more than 200 nt and a putative start codon (or a putative stop codon) were considered to be partial genes, which were characterized by a truncated open reading frame (ORF) resulting from either incomplete genome sequencing or poor genomic assembly; those with more than 200 nt and an interrupted reading frame were regarded to be pseudogenes. Fourth, we used newly obtained intact genes as queries to conduct TBLASTN searches against the genomes and attempted to identify additional *Tas2r*s. Fifth, all full-length genes were checked to predict whether the seven transmembrane domains were intact using the TMHMM method (Sonnhammer et al. 1998), and those without any of the domains were considered to be partial genes. We additionally assessed whether the partial genes are from independent loci or not, which is particularly necessary for low-coverage genomes. If multiple partial genes from a given species share a same orthology but do not overlap, these partial genes could be a single gene due to poor genomic assembly (supplementary table S2, Supplementary Material online), as suggested in a previous study (Hayakawa et al. 2014). Synteny analysis is also helpful to assess whether partial genes are unique, as shown in supplementary table S3, Supplementary Material online. All candidate genes were ultimately verified by BLASTN searches against the GenBank database, with the best hits being the known *Tas2r* genes. The deduced protein sequences of all newly identified intact genes are provided in the supplementary data set S1, Supplementary Material online.

### Phylogenetic Analysis

A total of 116 avian and 20 crocodilian intact *Tas2r*s were analyzed with an alligator *V1r1* gene (GenBank: XM_006031313) as the outgroup because vertebrate *V1r* genes are closely related to *Tas2r*s among the G protein-coupled receptor genes (Matsunami et al. 2000; Shi and Zhang 2006). The 136 *Tas2r*s and 1 *V1r1* were translated into protein sequences and were subsequently aligned with the MUSCLE program (Edgar 2004), and the resulting alignment was subjected to manual inspection in MEGA6 (Tamura et al. 2013). Phylogenetic analyses were conducted by both Neighbor-Joining (NJ) (Saitou and Nei 1987) and Bayesian Inference (BI) (Yang and Rannala 1997) approaches. The NJ phylogenetic tree was reconstructed using the protein Poisson distances (Nei and Kumar 2000) and the pairwise deletion of gap sites implemented in MEGA6 and was evaluated with 1,000 bootstrap replicates (Felsenstein 1985a). The BI tree was constructed by MrBayes version 3.1.2 (Ronquist and Huelsenbeck 2003) with 6 million generations after the best-fitting substitution model was determined by the jModelTest2 program (Darriba et al. 2012), following Bayesian information criterion (Posada and Buckley 2004).

### Evolutionary Analysis

To infer the processes of gains and losses of *Tas2r*s across the bird phylogeny, we carried out a reconciliation analysis in NOTUNG 2.6 program (Chen et al. 2007) by comparing the species tree with the gene tree. This method works with a nonbinary gene tree where some nodes are collapsed due to weak support. The gene gains and losses were predicted by the incongruence between the species and gene trees on the basis of the parsimony principle. The species tree topology was taken from a recent study (Zhang et al. 2014), while the gene tree topology was from our BI tree (supplementary fig. S1, Supplementary Material online) where nodes with Bayesian posterior probability below 50% were collapsed, as shown in the supplementary fig. S2, Supplementary Material online.

To determine the potential impact of the feeding ecology on the evolution of the *Tas2r* gene repertoire size in birds, we coded each bird as 0 (carnivore) and 1 (insectivore/folivore/frugivore/granivore/nectarivore) according to the abundance of plant products or insect tissues in their diets because plant and insect tissues may have the most abundant potential toxins, whereas noninsect animals have the least. With one exception, all the studied omnivorous birds were described in the relevant literature (supplementary table S1, Supplementary Material online). The diet of each omnivorous species appeared to contain 51% or more plant and insect tissues; we therefore coded each omnivorous bird as 1. The only exception is the carnivorous red-legged seriema (del Hoyo et al. 1992), for which we were unable to determine the amount of noninsect, insect, and plant tissues in its diet; we therefore coded the red-legged seriema as 0 and 1 separately to verify the analysis. A regression analysis of *Tas2r* gene repertoire size against diet codes was conducted. Because our data do not fit the standard normal distribution (*P* < 0.05, Kolmogorov–Smirnov test), the nonparametric Spearman’s rank correlation coefficient (ρ) was used to assess the correlation. As described earlier, we used two sets of *Tas2r* genes to test the consistency: The first consisted of all putatively functional *Tas2r*s (intact and partial genes), and the second comprised all *Tas2r*s (intact, partial, and pseudogenes). Functional genes can reflect the physiological needs, and identifiable pseudogenes that were recently lost may also reflect the physiological needs. Indeed, both the total number and the proportion of functional olfactory receptor genes were found to be positively correlated with olfactory acuity in mammals (Rouquier et al. 2000; Gilad et al. 2004). Because the phylogenetic inertia can potentially confound comparative analyses across a group of species (Fisher and Owens 2004), we performed a phylogenetically independent contrast (PIC) analysis implemented in the package Analyses of Phylogenetics and Evolution (Paradis et al. 2004). The input tree is the species tree (Zhang et al. 2014), and the branch lengths were estimated from the divergence times among species according to a recent study (Jarvis et al. 2014) and the TimeTree database (http://www.timetree.org/, last accessed June 30, 2015). We did not include the white-throated sparrow because its divergence time from other birds is unknown.

## Results

### Identification of *Tas2r*s

**Fig. 1.—:**
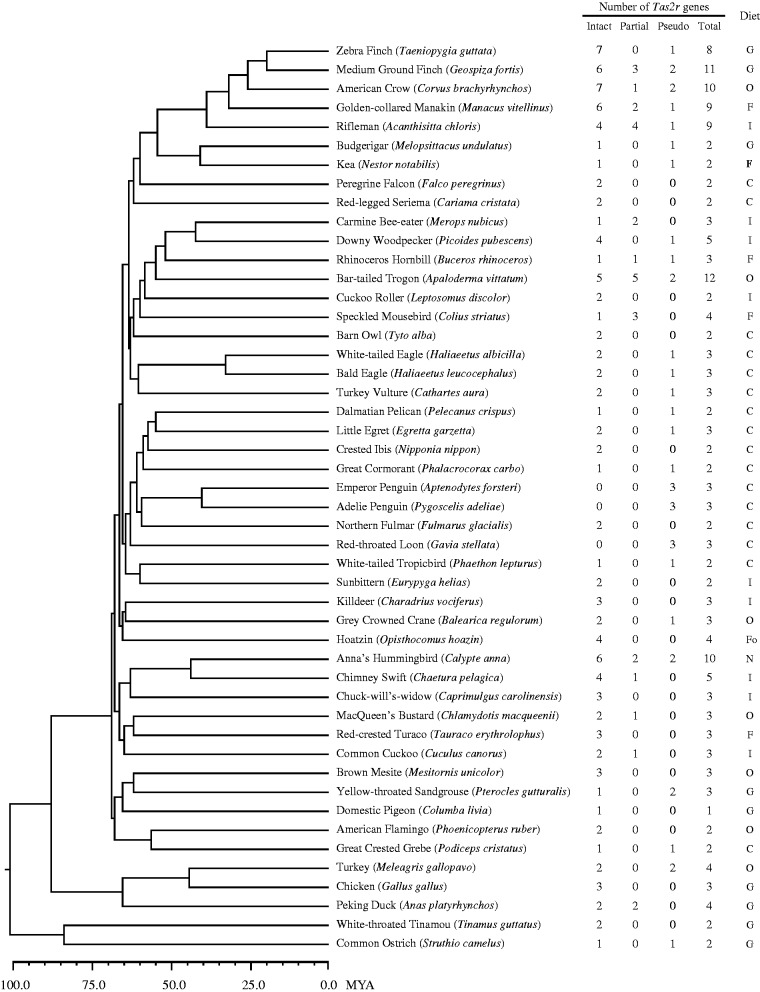
The bitter taste receptor gene repertoires of 48 birds and their dietary preferences. Species tree and divergence times were taken from a recent study (Jarvis et al. 2014). Dietary information was from the literature and the Animal Diversity Web (supplementary table S1, Supplementary Material online). C, carnivore; I, insectivore; F, frugivore; Fo, folivore; G, granivore; N, nectarivore; O, omnivore.

The avian *Tas2r* gene repertoire was characterized in few species due to the scarcity of available genome sequences (Li and Zhang 2014; Zhao et al. 2015). Recently, a total of 48 avian genome sequences were reported (Jarvis et al. 2014; Zhang et al. 2014). By using the published vertebrate *Tas2r*s as queries, we performed TBLASTN searches and identified *Tas2r*s from the genome sequences of 48 birds (fig. 1 and supplementary table S4, Supplementary Material online), representing all but three orders in the class Aves (Jarvis et al. 2014; Zhang et al. 2014). For convenience, we classified the identified *Tas2r*s into three categories: Intact genes (with an intact ORF and complete coding region), partial genes (with an intact ORF but partial coding region due to incomplete genome sequencing), and pseudogenes (with a disruptive ORF resulting from nonsense or frame-shifting mutations). The intact and partial genes are putatively functional, whereas the pseudogenes are possibly nonfunctional. We detected 0–7 intact genes (mean 2.4, median 2), 0–5 partial genes (mean 0.6, median 0), and 0–3 pseudogenes (mean 0.8; median 1) (fig. 1). The number of putatively functional *Tas2r*s in each species varied from 0 in the red-throated loon and the two penguins to 10 in the bar-tailed trogon, with a mean of 3 (fig. 1). Although all three categories of *Tas2r*s were counted, the gene number ranged from 1 in the domestic pigeon to 12 in the bar-tailed trogon, with an average of 4 (fig. 1). Overall, the *Tas2r* gene repertoire size in bird species is much smaller than that in mammals (Li and Zhang 2014). To detect whether avian *Tas2r*s resulted from tandem duplication as did mammalian *Tas2r*s, we checked the genomic location for each *Tas2r* gene. Indeed, some *Tas2r*s were found to be aligned in arrays (supplementary table S5, Supplementary Material online). Furthermore, we found that longer scaffolds tend to have more tandem duplicates of *Tas2r* genes (supplementary table S6, Supplementary Material online; *R* = 0.617, *P* = 0.025, Pearson correlation test), which is also a signature of tandem duplication. In addition, we similarly searched the genome sequences of 3 crocodilians, which are the closest outgroup of all extant birds, and identified 10, 6, and 11 *Tas2r*s (supplementary table S4, Supplementary Material online), suggesting that these reptiles have lower *Tas2r* gene numbers than other reptiles, such as the lizard (50 in total) (Li and Zhang 2014).

### Phylogenetic Reconstruction

**Fig. 2.—:**
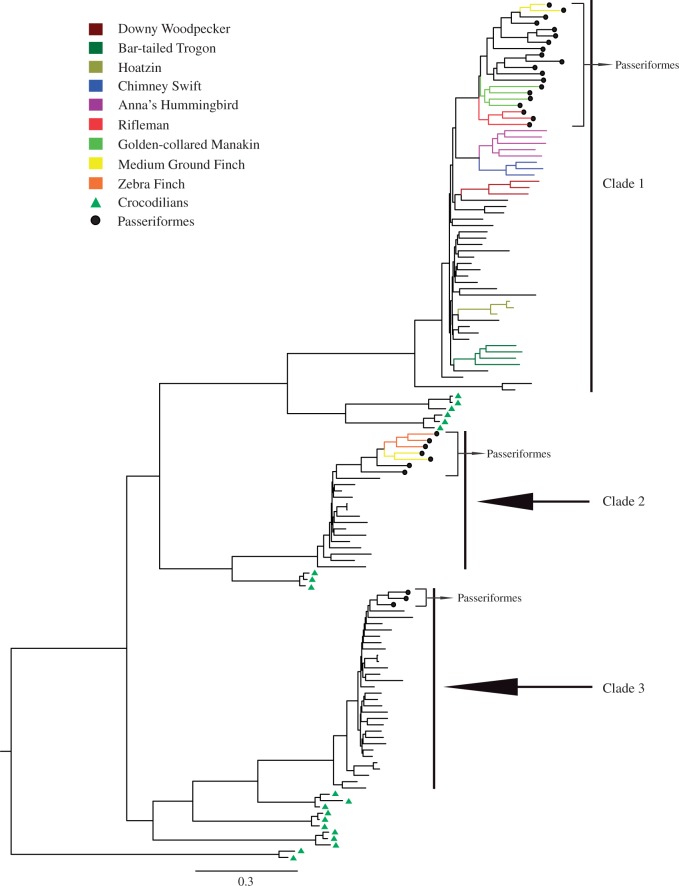
Evolutionary relationships of all 136 intact *Tas2r* genes from 48 birds and 3 crocodilians. The tree was reconstructed using the Bayesian approach with the best fitting model of GTR+I+G. Branch lengths were drawn to the scale. Putative species-specific gene duplications were marked in the branches with various colors, and members from Passeriformes were bracketed. The detailed information about species and gene names and Bayesian posterior probabilities was shown in supplementary fig. S1, Supplementary Material online, and the NJ tree showing a similar topology to this tree was provided in supplementary fig. S3, Supplementary Material online.

We aligned the deduced protein sequences of 136 intact *Tas2r*s from 45 birds (the red-throated loon and the 2 penguins have no intact *Tas2r*) and 3 crocodilians. The resulting alignment was used to construct phylogenetic trees with the NJ and BI approaches, and a crocodilian *V1r* gene was used as an outgroup. The partial genes and pseudogenes were not included in our phylogenetic analyses because most were too short to be aligned. The BI phylogenetic tree showed that all avian *Tas2r* genes formed three clades (fig. 2 and supplementary fig. S1, Supplementary Material online). Each of the three avian clades was allied with a group of crocodilian genes (fig. 2), suggesting that these genes appeared to have originated prior to the divergence of archosaurs (including crocodilians, dinosaurs, and birds). The first clade of avian genes was found to be enriched with putatively species-specific duplications, which are indicated by various colors (fig. 2). For example, the bar-tailed trogon has a cluster of four genes and Anna’s hummingbird is characterized by a cluster of five genes. Tests of gene conversion among paralogous genes were conducted using Sawyer’s method, as implemented in the software GENECONV (Sawyer 1989). Only two possible events of gene conversion were detected (supplementary table S7, Supplementary Material online), suggesting that such events may not have played a major role in avian *Tas2r* evolution. In addition, avian species from clades 2 and 3 have no species-specific gene duplications, except the medium ground finch and zebra finch (fig. 2). After removing gaps with the pairwise-deletion option, a total of 338 informative positions were used to build NJ tree. The NJ tree shows an overall topology similar to the BI tree (supplementary fig. S2, Supplementary Material online), although many more nodes of NJ tree were weakly supported. For comparison, we also selected the complete deletion option to remove gaps in building the NJ tree, and a total of 210 codons were used. Both deletion options resulted in nearly identical tree topologies (supplementary figs. S3 and S4, Supplementary Material online).

### Evolution of the *Tas2r* Gene Repertoire

**Fig. 3.—:**
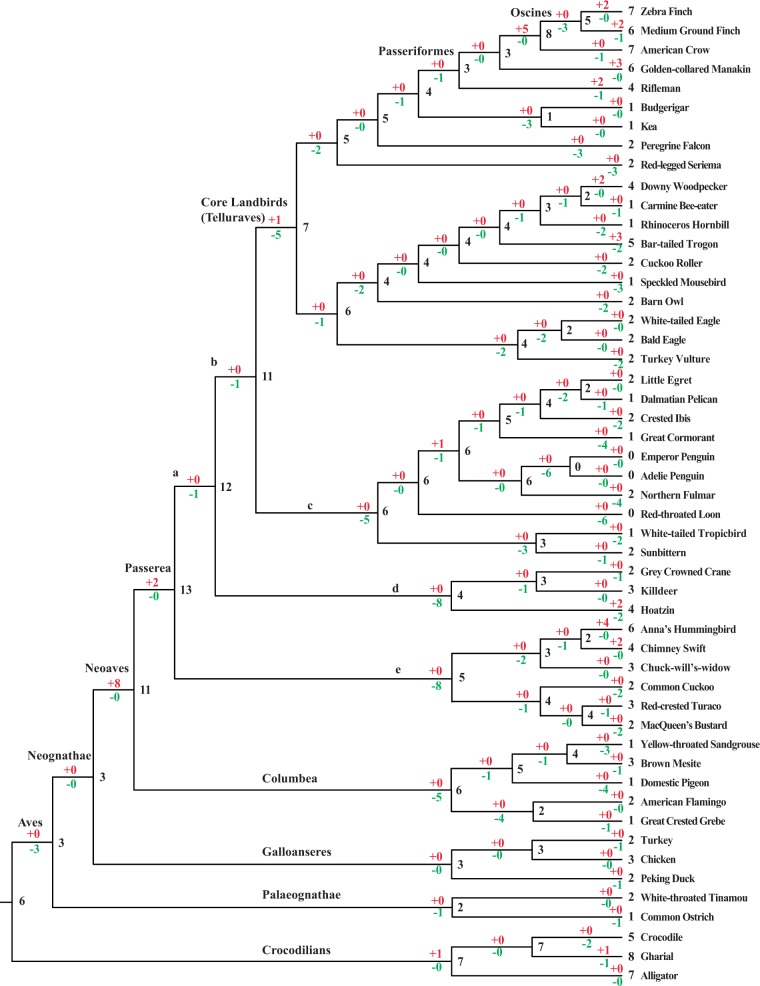
Evolutionary changes of intact *Tas2r* gene numbers in 48 birds and 3 crocodilians. The estimated *Tas2r* gene numbers for ancestral lineages were shown with black, whereas the numbers of gene gains and gene losses were indicated with purple and green, respectively.

To recover the evolutionary history of intact *Tas2r* gene repertoire among avian species, we predicted the numbers of intact *Tas2r*s in avian ancestors and inferred the evolutionary changes of intact *Tas2r* gene numbers in the ancestral and extant species by comparing the gene tree with the species tree using the reconciliation analysis (Chen et al. 2007). We used the BI tree (fig. 2 and supplementary fig. S1, Supplementary Material online) to predict gene number changes, because it appears to be better supported than the NJ tree (supplementary fig. S3, Supplementary Material online). Weakly supported branches with Bayesian posterior probability below 50% were collapsed, and the resulting gene tree was shown in supplementary fig. S2, Supplementary Material online. We found that the number of intact *Tas2r*s (6 genes) in the common ancestor of birds and crocodilians was small (fig. 3). Because a reduction occurred in the turtle (11 intact *Tas2r*s) compared with the lizard (36 intact *Tas2r*s) (Li and Zhang 2014), our data suggested that the reduction of *Tas2r*s may have occurred before the divergence between turtles and archosaurs (including crocodilians, dinosaurs, and birds) approximately 265 Ma (Janke and Arnason 1997; Green et al. 2014). Moreover, we observed a further reduction (*n* = 3) in the ancestral branch of all extant birds, which resulted in only three intact *Tas2r*s in the common ancestor of birds (fig. 3). The majority of ancestral lineages of birds carried a small intact *Tas2r* gene repertoire, while the branch a, branch b, and lineages leading to Neoaves and Passerea were estimated to have an intact gene number exceeding 10 (fig. 3). Substantial reductions (*n* ≥ 5) were observed in the lineage leading to Telluraves, branch c, branch d, branch e, and lineages leading to Columbea, the red-throated loon, and the common ancestor of penguins. In contrast, a substantial gene gain (*n* = 5) was inferred in the lineage leading to Oscines, suggestive of a slightly larger number of intact *Tas2r*s in Oscines compared with other birds (fig. 3). Evolutionary changes of the *Tas2r* gene number in chickens were controversial, with both an extensive gene loss (Go 2006) and no change (Dong et al. 2009) being proposed. In our analysis, a gene number change was not observed in chickens since their separation from turkeys (fig. 3).

**Fig. 4.—:**
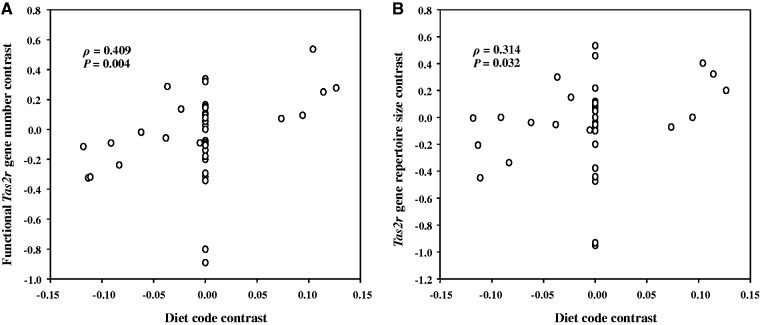
Dietary preferences impact the avian *Tas2r* gene repertoires. (*A*) PIC in putatively functional *Tas2r* gene number is positively correlated with that in diet preference; (*B*) PIC in total *Tas2r* gene number remains an increasing trend as PIC in diet codes increases, although it was only marginally significant. According to the amount of potential toxins in its diet, each bird was coded as 0 (carnivore), 1 (folivore), 1 (insectivore), 1 (frugivore), 1 (granivore), 1 (nectarivore), and 1 (omnivore). The Spearman’s rank correlation coefficient (ρ) with a two-tailed *P* value was used to evaluate the association.

To examine whether dietary preferences influenced the evolution of avian *Tas2r* gene repertoires, which are generally small in size, we divided birds into carnivores, insectivores, folivores, frugivores, granivores, nectarivores, and omnivores (fig. 1) according to the Animal Diversity Web (http://animaldiversity.org/, last accessed June 30, 2015) and other references (supplementary table S1, Supplementary Material online). Because insectivorous birds feed on insects, of which many species release defensive secretions that are toxic to their predators (Weatherston and Percy 1970; Blum 1981; de Jong et al. 1991), we assumed that insectivorous birds may confront a similar amount of toxins as their herbivorous relatives, although it is well known that plant tissues tend to contain more toxins than animal tissues (Glendinning 1994; Wang et al. 2004; Li and Zhang 2014). Indeed, this assumption was supported by multiple *Tas2r* gene expansions in insect-eating bats rather than fruit-eating bats (Zhou et al. 2009). We predicted that carnivorous birds carry smaller *Tas2r* gene repertoires than other birds. We coded the dietary preference in a bird as 0 (carnivore) or 1 (insectivore/folivore/frugivore/granivore/nectarivore) under the assumption that other birds consuming more plant and insect tissues encounter more toxins than do carnivorous birds. After converting the diet codes and the *Tas2r* gene numbers into PICs (Felsenstein 1985b), we conducted a regression analysis. We observed a significant positive correlation between the PICs of the functional *Tas2r* gene numbers and those of diet codes (Spearman’s ρ = 0.409, *P* = 0.004; fig. 4). The same trend was revealed when the PICs of the total *Tas2r* numbers were correlated with the PICs of the diet codes (ρ = 0.314, *P* = 0.032; fig. 4). We repeated the PIC analysis while coding the red-legged seriema as 1 because this bird may be omnivorous (del Hoyo et al. 1992). The repeated analysis confirmed the correlation between diet codes and functional *Tas2r* gene numbers (ρ = 0.335, *P* = 0.021; supplementary fig. S5, Supplementary Material online) and revealed a same trend between the PICs of diet codes and those of total *Tas2r* gene numbers, although it was not significant (ρ = 0.235, *P* = 0.111; supplementary fig. S5, Supplementary Material online). To compare with an earlier vertebrate-wide study (Li and Zhang 2014), we also coded insectivores as 0, the positive correlation between the PICs of diet codes and those of functional gene numbers remains significant (ρ = 0.319, *P* = 0.029). Our findings clearly showed a significant positive correlation between the number of functional *Tas2r*s in birds and the amount of potential toxins in their diet.

## Discussion

With the advent of 45 recently released avian genome sequences, we identified 215 *Tas2r*s from 48 avian and 3 crocodilian genomes and characterized the evolutionary history of avian *Tas2r*s spanning approximately 100 My (Jarvis et al. 2014). The avian *Tas2r* gene repertoire contains approximately 4 members, on average, ranging from 1 to 12 (fig. 1 and supplementary table S4, Supplementary Material online). Relative to most other vertebrates (Li and Zhang 2014), bird species exhibit a dramatic reduction in the *Tas2r* repertoire size. Furthermore, we found that carnivorous birds carry a smaller *Tas2r* repertoire than do other birds and observed a positive correlation between the number of putatively functional *Tas2r*s and the amount of potential toxins in the diet, supporting the hypothesis that dietary toxins have driven the evolution of bitter taste receptor genes, even in bird species carrying diminutive *Tas2r* repertoires.

The 48 avian species with whole genome sequences represent all but 3 orders of birds (Zhang et al. 2014), providing an excellent opportunity to recover an overall evolutionary history of *Tas2r*s across bird species. Our study unambiguously revealed a general pattern that bird species carry a small *Tas2r* gene repertoire relative to other vertebrates (Li and Zhang 2014). This finding is consistent with anatomical evidence, which showed fewer taste buds and a lower number of taste receptors in bird species compared with other vertebrates (Berkhoudt 1985; Mason and Clark 2000). Notably, the red-throated loon, Adelie penguin, and emperor penguin possess no functional *Tas2r*, which suggested a loss of bitter taste perception. Other than the 3 bird species mentioned, the remaining bird species carry at least one functional *Tas2r*, indicating that the bitter taste function is retained in 45 birds. We also observed a few gene clusters consisting of 2–5 genes, but we did not detect a large expansion in any bird comparable with the white-throated sparrow (*Z. albicollis*), which was found to possess a gene cluster encoding 18 functional Tas2r receptors (Davis et al. 2010). The lineage-specific expansion in the white-throated sparrow may not be an isolated case because we identified a gene gain (*n* = 5) in the ancestral lineage of Oscines (fig. 3). Indeed, we observed that the five passerine birds studied carry a larger *Tas2r* repertoire compared with most other bird species (fig. 1). The varying coverage of genome sequences (Zhang et al. 2014) may affect gene identification, but it is not the case for these avian genomes because we identified a low number of *Tas2r*s from each avian genome, irrespective of the genome coverage. Our additional analysis did not detect a correlation between the fraction of partial genes and contig N50 length (ρ = 0.141, *P* = 0.374; supplementary fig. S6, Supplementary Material online), possibly because birds typically have fewer partial *Tas2r* genes (mean 0.6, median 0) and most birds (35 out of 48) have no partial *Tas2r*s (fig. 1). However, the small *Tas2r* repertoires in birds do not necessarily mean a reduced importance of bitter taste, which could be compensated for by either the recognition of more bitter compounds or the development of novel taste receptors. For example, all chicken and turkey Tas2r receptors were able to recognize a wide range of bitter chemicals (Behrens et al. 2014); the hummingbird repurposed the ancestral umami receptor to compensate for the loss of *Tas1r2*, which encodes a canonical sweet receptor (Zhao et al. 2003; Baldwin et al. 2014). Despite this, birds appear to have a less developed sense of bitter taste than mammals, as a higher number of *Tas2r*s allows the evolution of more specialized bitter taste receptors (Behrens et al. 2014).

Evolution of the narrow *Tas2r* gene repertoires in birds still reflects the changes in dietary preferences, with a positive correlation between the functional *Tas2r* gene number and abundance of potential toxins in the diet. Because herbivorous birds consume plant products that typically contain more toxins than animal tissues and insectivorous birds feed on insects that may release defensive secretions toxic to birds, both herbivorous and insectivorous birds are expected to require more *Tas2r*s than carnivorous birds eating noninsect animals. Our present findings appear to support the expectation that dietary toxins shaped the *Tas2r* gene repertoires in birds. In addition, we also observed some cases of discrepancies between the gene number and food habit. For example, three birds clearly have a diet consisting of potential toxins, yet have only 1 intact *Tas2r* and 3 in total (carmine bee-eater), and 2 intact *Tas2r*s and 3 in total (common cuckoo). These discrepancies may result from the narrowness of their diets, as proposed in vampire bats (Zhao et al. 2010; Hong and Zhao 2014). Future studies are needed to evaluate other ecological factors that are potentially involved.

Consistent with the observation across all of the vertebrates examined (Li and Zhang 2014), we observed a similar pattern in birds, a subgroup of vertebrates, suggesting that diet impacts *Tas2r* evolution at both large and small scales. It would be interesting to measure, at a smaller scale, the tuning properties of Tas2r receptors in populations or closely related species with variations in bitter taste ability. In contrast, a larger genome size cannot predict more *Tas2r*s in birds because all birds examined have similar genome sizes, ranging from 1.05 to 1.26 Gb (Zhang et al. 2014). However, other than diet, additional driving forces must be involved in shaping *Tas2r* diversity. For instance, all but one *Tas2r*s were pseudogenes in both toothed and baleen whales (Feng et al. 2014; Kishida et al. 2015), possibly driven by the high concentration of sodium in the ocean, the feeding behavior of swallowing food whole, and the dietary switch from plants to meat in ancient whales (Feng et al. 2014); while the two *Tas2r*s are intact in their outgroup species, both genes were pseudogenized in the common ancestor of all extant penguins, which may result from the extremely cold Antarctic (Zhao et al. 2015). All modern bird species lack teeth and swallow food without mastication (Meredith et al. 2014), and hence, this feeding behavior should not account for the *Tas2r* evolution in the case of bird species. In addition to diet selection, however, extraoral functions (e.g., in gastrointestinal tract or respiratory epithelium) (Wu et al. 2002; Finger et al. 2003) may also drive the evolution of bitter taste receptor genes in birds, a hypothesis that awaits future investigation.

## Supplementary Material

Supplementary figures S1–S6, tables S1–S7, and data set S1 are available at *Genome Biology and Evolution* online (http://www.gbe.oxfordjournals.org/).

Supplementary Data
