# Supplementary material for: Birds Generally Carry a Small Repertoire of Bitter Taste Receptor Genes
Source: Genome Biol Evol. 2015 Sep 4;7(9):2705–15. doi: 10.1093/gbe/evv180 (PMC4607536; doi:10.1093/gbe/evv180)
Supplement: Supplementary Data [file supp_7_9_2705__index.html]

Birds Generally Carry a Small Repertoire of Bitter Taste Receptor Genes — Supplementary Data 

# Birds Generally Carry a Small Repertoire of Bitter Taste Receptor Genes

## Supplementary Data

files

- Supplementary Data - pdf file
