# Supplementary material for: Birds Generally Carry a Small Repertoire of Bitter Taste Receptor Genes
Source: Genome Biol Evol. 2015 Sep 4;7(9):2705–15. doi: 10.1093/gbe/evv180 (PMC4607536; doi:10.1093/gbe/evv180)
Supplement: Supplementary Data [file supp_evv180_Supplementary_File.pdf]

Supplementary Information for

Birds generally carry a small repertoire of bitter taste receptor genes

Authors:

Kai Wang, and Huabin Zhao* (E-mail: huabinzhao@whu.edu.cn)

Affiliation:

Department of Ecology, College of Life Sciences, Wuhan University, Wuhan, China

This PDF file contains:

Table S1 to S7

Figures S1 to S6

Data set S1

Table S1. References of the dietary preferences and genomic contig N50 statistics for the 48 birds studied.

Species	Contig N50 (bp)	Diet	References*
Rifleman	18K	Insectivore	Oliver 1955
Golden-collared Manakin	34K	Frugivore	Worthington 1989
Medium Ground Finch	30K	Granivore	Scott 1996
American Crow	24K	Omnivore	http://animaldiversity.org
Zebra Finch	38K	Granivore	http://animaldiversity.org
Budgerigar	55K	Granivore	DeGolier et al. 1999
Kea	16K	Frugivore	Brejaart 1988
Peregrine Falcon	28K	Carnivore	http://animaldiversity.org
Red-legged Seriema	17K	Carnivore	del Hoyo et al. 1992
Carmine Bee-eater	20K	Insectivore	Hoi et al. 2015
Downy Woodpecker	20K	Insectivore	http://animaldiversity.org
Rhinoceros Hornbill	14K	Frugivore	Kemp and Woodcock. 1995
Bar-tailed Trogon	19K	Omnivore	del Hoyo et al. 1992
Cuckoo Roller	19K	Insectivore	del Hoyo et al. 1992
Speckled Mousebird	18K	Frugivore	Roberts et al, 2005
Barn Owl	13K	Carnivore	DeGolier et al, 1999
White-tailed Eagle	20K	Carnivore	del Hoyo et al. 1992
Bald Eagle	105K	Carnivore	http://animaldiversity.org
Turkey Vulture	12K	Carnivore	http://animaldiversity.org
Dalmatian Pelican	18K	Carnivore	del Hoyo et al. 1992
Little Egret	24K	Carnivore	Goutner et al. 1997
Crested Ibis	22K	Carnivore	del Hoyo et al. 1992
Great Cormorant	15K	Carnivore	http://animaldiversity.org
Northern Fulmar	17K	Carnivore	http://animaldiversity.org
Emperor Penguin	30K	Carnivore	http://animaldiversity.org
Adelie Penguin	19K	Carnivore	http://animaldiversity.org
Red-throated Loon	16K	Carnivore	http://animaldiversity.org
White-tailed Tropicbird	18K	Carnivore	Schreiber. 1991
Sunbittern	16K	Insectivore	Knowlton. 1909
Killdeer	32K	Insectivore	DeGolier et al. 1999
Grey Crowned Crane	18K	Omnivore	http://animaldiversity.org
Hoatzin	24K	Folivore	http://animaldiversity.org
Anna’s Hummingbird	23K	Nectarivore	DeGolier et al. 1999
Chimney Swift	28K	Insectivore	http://animaldiversity.org
Chuck-will's-widow	22K	Insectivore	http://animaldiversity.org
MacQueen's Bustard	18K	Omnivore	Perrins. 2009
Red-crested Turaco	18K	Frugivore	del Hoyo et al. 1992
Common Cuckoo	31K	Insectivore	http://animaldiversity.org
Brown Mesite	18K	Omnivore	del Hoyo et al. 1992
Yellow-throated Sandgrouse	26K	Granivore	Campbell and Lack. 1985

Domestic Pigeon	22K	Granivore	DeGolier et al. 1999
American Flamingo	16K	Omnivore	Perrins 2009
Great Crested Grebe	13K	Carnivore	http://animaldiversity.org
Turkey	26K	Omnivore	DeGolier et al. 1999
Chicken	279K	Granivore	DeGolier et al. 1999
Peking Duck	26K	Granivore	DeGolier et al. 1999
White-throated Tinamou	24K	Granivore	Perrins 2009
Common Ostrich	29K	Granivore	http://animaldiversity.org
Alligator		Carnivore	http://animaldiversity.org
Crocodile		Carnivore	http://animaldiversity.org
Gharial		Carnivore	http://animaldiversity.org

*References cited.

Brejaart, R. 1988 Diet and feeding behaviour of the kea (*Nestor notabilis*). Lincoln, Lincoln University.

Campbell, B., Lack, E. & British Ornithologists' Union. 1985 A dictionary of birds. Vermillion, S.D., Published for the British Ornithologists' Union by Buteo Books.

DeGolier, T.F., Mahoney, S.A. & Duke, G.E. 1999 Relationships of avian cecal lengths to food habits, taxonomic position, and intestinal lengths. Condor 101, 622-634.

del Hoyo, J., Elliott, A., Sargatal, J. & Cabot, J. 1992 Handbook of the birds of the world. Barcelona, Lynx Edicions.

Goutner, V. & Furness, R.W. 1997 Mercury in feathers of little egret *Egretta garzetta* and night heron *Nycticorax nycticorax* chicks and in their prey in the Axios Delta, Greece. Arch. Environ. Contam. Toxicol. 32, 211-216.

Hoi, H., Kristofik, J. & Darolova, A. 2015 All you can eat: is food supply unlimited in a colonially breeding bird? Ecol Evol 5, 450-458.

Kemp, A.C. & Woodcock, M. 1995 The hornbills: Bucerotiformes. New York, Oxford University Press.

Knowlton, F.H. & Lucas, F.A. 1909 Birds of the World: A popular account, Holt.

Oliver, W.R.B. 1955 New Zealand birds. 2nd ed. Wellington, AH & AW Reed.

Perrins, C.M. 2009 The Princeton encyclopedia of birds. Princeton, N.J., Princeton University Press.

Roberts, A., Hockey, P.A.R., Dean, W.R.J. & Ryan, P. 2005 Roberts' birds of Southern Africa. 7th ed. Cape Town, Trustees of the John Voelcker Bird Book Fund.

Schreiber, E.A. 1991 Encyclopaedia of Animals: Birds. London, Merehurst Press.

Scott, T.A. 1996 Concise encyclopedia biology. Berlin, Walter de Gruyter.

Worthington, A. 1989 Adaptations for avian frugivory: assimilation efficiency and gut transit time of *Manacus vitellinus* and *Pipra mentalis.* Oecologia 80, 381-389.

Table S2. All partial *Tas2r* genes identified in this study. Non-overlapping partial genes (shown in bold) in one species with similar orthology are potentially from the same locus with gaps.

Species/Genome	Different orthologs	Non-overlapping *Tas2r*s potentially from same locus
Rifleman	Rifleman_Tas2r5_P<br>Rifleman_Tas2r6_P<br>Rifleman_Tas2r7_P<br>Rifleman_Tas2r8_P	None
Golden-collared Manakin	Golden-collared_Manakin_Tas2r7_P<br>Golden-collared_Manakin_Tas2r8_P	None
Medium Ground Finch	Medium_Ground_Finch_Tas2r9_P<br>Medium_Ground_Finch_Tas2r7_P<br>Medium_Ground_Finch_Tas2r8_P	None
Carmine Bee-eater	Carmine_Bee-eater_Tas2r4_P	**Carmine_Bee-eater_Tas2r2_P**<br>**Carmine_Bee-eater_Tas2r3_P**
Bar-tailed Trogon	Bar-tailed_Trogon_Tas2r6_P<br>Bar-tailed_Trogon_Tas2r8_P<br>Bar-tailed_Trogon_Tas2r9_P<br>Bar-tailed_Trogon_Tas2r11_P	**Bar-tailed_Trogon_Tas2r7_P**<br>**Bar-tailed_Trogon_Tas2r10_P**
Speckled Mousebird	Speckled_Mousebird_Tas2r2_P<br>Speckled_Mousebird_Tas2r3_P<br>Speckled_Mousebird_Tas2r4_P	None
Anna’s Hummingbird	Hummingbird_Tas2r8_P	**Hummingbird_Tas2r7_P**<br>**Hummingbird_Tas2r9_P**<br>**Hummingbird_Tas2r10_P**
Chimney Swift	None	**Chimney_Swift_Tas2r6_P**<br>**Chimney_Swift_Tas2r5_P**
MacQueen's Bustard	None	**MacQueens_Bustard_Tas2r3_P**<br>**MacQueens_Bustard_Tas2r4_P**
Peking Duck	Peking_Duck_Tas2r3_P<br>Peking_Duck_Tas2r4_P	None

Table S3. Syntenic analysis for partial genes.

Species	Gene name	Scaffold	Upstream gene	Downstream gene
Rifleman	Rifleman_Tas2r5_P	scaffold12242	poly N (incomplete sequencing)	taste receptor type 2 member 9-like
	Rifleman_Tas2r6_P	scaffold28229	NA (No available data due to short contig)	NA
	Rifleman_Tas2r7_P	C16332196	No blast hit	NA
	Rifleman_Tas2r8_P	scaffold28228	NA	NA
Golden-collared Manakin	Golden-collared_Manakin_Tas2r7_P	scaffold920	meprin A subunit alpha	NA
	Golden-collared_Manakin_Tas2r8_P	scaffold2273	NA	No blast hit
Medium Ground Finch	Medium_Ground_Finch_Tas2r9_P	scaffold1047	taste receptor type 2 member 40-like	poly N
	Medium_Ground_Finch_Tas2r7_P	scaffold4119	NA	NA
	Medium_Ground_Finch_Tas2r8_P	scaffold748	poly N	No blast hit
Carmine Bee-eater	Carmine_Bee-eater_Tas2r2_P	scaffold19532	meprin A subunit alpha	NA
	Carmine_Bee-eater_Tas2r3_P	scaffold20440	NA	NA
	Carmine_Bee-eater_Tas2r4_P	scaffold41834	NA	No blast hit
Bar-tailed Trogon	Bar-tailed_Trogon_Tas2r6_P	scaffold6731	poly N	No blast hit
	Bar-tailed_Trogon_Tas2r7_P	scaffold2738	poly N	transposon-derived protein ZK1236.4
	Bar-tailed_Trogon_Tas2r8_P	scaffold23126	NA	No blast hit
	Bar-tailed_Trogon_Tas2r9_P	scaffold15085	NA	poly N
	Bar-tailed_Trogon_Tas2r10_P	scaffold22121	poly N	NA
	Bar-tailed_Trogon_Tas2r11_P	scaffold41964	NA	NA
Speckled Mousebird	Speckled_Mousebird_Tas2r2_P	scaffold37637	NA	NA
	Speckled_Mousebird_Tas2r3_P	scaffold38140	No blast hit	poly N
	Speckled_Mousebird_Tas2r4_P	scaffold37670	No blast hit	poly N
Anna’s Hummingbird	Hummingbird_Tas2r7_P	C10465731	NA	NA
	Hummingbird_Tas2r8_P	C10690480	NA	NA

	Hummingbird_Tas2r9_P	C10499692	NA	NA
	Hummingbird_Tas2r10_P	C10429533	NA	NA
Chimney Swift	Chimney_Swift_Tas2r6_P	scaffold99	Taste receptor type 2 member 9	poly N
	Chimney_Swift_Tas2r5_P	scaffold46	poly N	transposon-derived protein ZK1236.4
MacQueen's Bustard	MacQueens_Bustard_Tas2r3_P	C15284394	NA	NA
	MacQueens_Bustard_Tas2r4_P	C15304738	No blast hit	NA
Peking Duck	Peking_Duck_Tas2r3_P	C18799681	NA	NA
	Peking_Duck_Tas2r4_P	scaffold2869	NA	ankyrin repeat domain-containing protein

Table S4. *Tas2r* gene repertoires of 48 birds and three crocodilians examined.

Order	Common name	Species name	Diet*	Number of *Tas2r* genes			
				Intact	Partial	Pseudo	Total
**Passeriformes**	Rifleman	*Acanthisitta chloris*	Insectivore	4	4	1	9
	Golden-collared Manakin	*Manacus vitellinus*	Frugivore	6	2	1	9
	Medium Ground Finch	*Geospiza fortis*	Granivore	6	3	2	11
	American Crow	*Corvus brachyrhynchos*	Omnivore	7	1	2	10
	Zebra Finch	*Taeniopygia guttata*	Granivore	7	0	1	8
**Psittaciformes**	Budgerigar	*Melopsittacus undulatus*	Granivore	1	0	1	2
	Kea	*Nestor notabilis*	Frugivore	1	0	1	2
**Falconiformes**	Peregrine Falcon	*Falco peregrinus*	Carnivore	2	0	0	2
**Cariamiformes**	Red-legged Seriema	*Cariama cristata*	Omnivore	2	0	0	2
**Coraciiformes**	Carmine Bee-eater	*Merops nubicus*	Insectivore	1	2	0	3
**Piciformes**	Downy Woodpecker	*Picoides pubescens*	Insectivore	4	0	1	5
**Bucerotiformes**	Rhinoceros Hornbill	*Buceros rhinoceros*	Frugivore	1	1	1	3
**Trogoniformes**	Bar-tailed Trogon	*Apaloderma vittatum*	Omnivore	5	5	2	12
**Leptosomiformes**	Cuckoo Roller	*Leptosomus discolor*	Insectivore	2	0	0	2
**Coliiformes**	Speckled Mousebird	*Colius striatus*	Frugivore	1	3	0	4
**Strigiformes**	Barn Owl	*Tyto alba*	Carnivore	2	0	0	2
**Accipitriformes**	White-tailed Eagle	*Haliaeetus albicilla*	Carnivore	2	0	1	3
	Bald Eagle	*Haliaeetus leucocephalus*	Carnivore	2	0	1	3
	Turkey Vulture	*Cathartes aura*	Carnivore	2	0	1	3
**Pelecaniformes**	Dalmatian Pelican	*Pelecanus crispus*	Carnivore	1	0	1	2
	Little Egret	*Egretta garzetta*	Carnivore	2	0	1	3
	Crested Ibis	*Nipponia nippon*	Carnivore	2	0	0	2
	Great Cormorant	*Phalacrocorax carbo*	Carnivore	1	0	1	2
**Procellariiformes**	Northern Fulmar	*Fulmarus glacialis*	Carnivore	2	0	0	2
**Sphenisciformes**	Emperor Penguin	*Aptenodytes forsteri*	Carnivore	0	0	3	3
	Adelie Penguin	*Pygoscelis adeliae*	Carnivore	0	0	3	3
**Gaviiformes**	Red-throated Loon	*Gavia stellata*	Carnivore	0	0	3	3
**Phaethontiformes**	White-tailed Tropicbird	*Phaethon lepturus*	Carnivore	1	0	1	2
**Eurypygiformes**	Sunbittern	*Eurypyga helias*	Insectivore	2	0	0	2
**Charadriiformes**	Killdeer	*Charadrius vociferus*	Insectivore	3	0	0	3
**Gruiformes**	Grey Crowned Crane	*Balearica regulorum*	Omnivore	2	0	1	3
**Opisthocomiformes**	Hoatzin	*Opisthocomus hoazin*	Folivores	4	0	0	4
**Caprimulgiformes**	Anna’s Hummingbird	*Calypte anna*	Nectarivore	6	2	2	10
	Chimney Swift	*Chaetura pelagica*	Insectivore	4	1	0	5
	Chuck-will's-widow	*Caprimulgus carolinensis*	Insectivore	3	0	0	3
**Otidiformes**	MacQueen's Bustard	*Chlamydotis macqueenii*	Omnivore	2	1	0	3
**Musophagiformes**	Red-crested Turaco	*Tauraco erythrolophus*	Frugivore	3	0	0	3
**Cuculiformes**	Common Cuckoo	*Cuculus canorus*	Insectivore	2	1	0	3
**Mesitornithiformes**	Brown Mesite	*Mesitornis unicolor*	Omnivore	3	0	0	3
**Pterocliformes**	Yellow-throated Sandgrouse	*Pterocles gutturalis*	Granivore	1	0	2	3
**Columbiformes**	Domestic Pigeon	*Columba livia*	Granivore	1	0	0	1

**Phoenicopteriformes**	American Flamingo	*Phoenicopterus ruber*	Omnivore	2	0	0	2
**Podicipediformes**	Great Crested Grebe	*Podiceps cristatus*	Carnivore	1	0	1	2
**Galliformes**	Turkey	*Meleagris gallopavo*	Omnivore	2	0	2	4
	Chicken	*Gallus gallus*	Granivore	3	0	0	3
**Anseriformes**	Peking Duck	*Anas platyrhynchos*	Granivore	2	2	0	4
**Tinamiformes**	White-throated Tinamou	*Tinamus guttatus*	Granivore	2	0	0	2
**Struthioniformes**	Common Ostrich	*Struthio camelus*	Granivore	1	0	1	2
**Crocodylia**	Alligator	*Alligator mississippiensis*	Carnivore	7	0	3	10
	Crocodile	*Crocodylus porosus*	Carnivore	5	0	1	6
	Gharial	*Gavialis gangeticus*	Carnivore	8	0	3	11

[*] Note - The diet classification for each bird was determined by a food type predominated in its diet with 51% or more.

Table S5. Genomic locations for all avian *Tas2r* genes studied. Potential tandem duplicated genes were highlighted in gray.

Species	Gene name	Gene type	Genomic location			
			Scaffold	Subject start	Subject end	Orientation
Rifleman	Rifleman_Tas2r2	Intact	scaffold4455	7457	6453	-
	Rifleman_Tas2r1	Intact	scaffold4455	2206	1253	-
	Rifleman_Tas2r3	Intact	scaffold8056	12904	11933	-
	Rifleman_Tas2r5_P	Partial	scaffold12242	3985	4458	+
	Rifleman_Tas2r4	Intact	scaffold12242	7500	8450	+
	Rifleman_Tas2r6_P	Partial	scaffold28229	1	684	+
	Rifleman_Tas2r7_P	Partial	C16332196	463	681	+
	Rifleman_Tas2r8_P	Partial	scaffold28228	1	681	+
	Rifleman_Tas2r9_Ps	Pseudogene	scaffold31809	1100	1402	+
Golden-collared Manakin	Golden-collared_Manakin_Tas2r1	Intact	scaffold208	227981	227016	-
	Golden-collared_Manakin_Tas2r3	Intact	scaffold300	1025772	1024837	-
	Golden-collared_Manakin_Tas2r2	Intact	scaffold114	1187	2152	+
	Golden-collared_Manakin_Tas2r9_Ps	Pseudogene	scaffold114	6615	6999	+
	Golden-collared_Manakin_Tas2r6	Intact	scaffold114	9190	10137	+
	Golden-collared_Manakin_Tas2r4	Intact	scaffold114	14217	15158	+
	Golden-collared_Manakin_Tas2r5	Intact	scaffold114	17745	18698	+
	Golden-collared_Manakin_Tas2r7_P	Partial	scaffold920	8287	8942	+
	Golden-collared_Manakin_Tas2r8_P	Partial	scaffold2273	588	76	-
Medium Ground Finch	Medium_Ground_Finch_Tas2r1	Intact	scaffold52	273539	272586	-
	Medium_Ground_Finch_Tas2r3	Intact	scaffold52	268377	267424	-
	Medium_Ground_Finch_Tas2r4	Intact	scaffold52	265312	264353	-
	Medium_Ground_Finch_Tas2r2	Intact	scaffold884	340322	339363	-
	Medium_Ground_Finch_Tas2r9_P	Partial	scaffold1047	222842	222346	-
	Medium_Ground_Finch_Tas2r5	Intact	scaffold1047	226160	227095	+
	Medium_Ground_Finch_Tas2r6	Intact	scaffold1047	229180	230115	+
	Medium_Ground_Finch_Tas2r7_P	Partial	scaffold4119	1	474	+

	Medium_Ground_Finch_Tas2r8_P	Partial	scaffold748	1173	218	-
	Medium_Ground_Finch_Tas2r10_Ps	Pseudogene	scaffold16	15333977	15334638	+
	Medium_Ground_Finch_Tas2r11_Ps	Pseudogene	scaffold98	2326511	2326828	+
American Crow	American_Crow_Tas2r5	Intact	scaffold116	14005127	14005987	+
	American_Crow_Tas2r4	Intact	scaffold116	14009670	14010503	+
	American_Crow_Tas2r6	Intact	scaffold116	14014569	14015522	+
	American_Crow_Tas2r3	Intact	scaffold116	14020521	14021474	+
	American_Crow_Tas2r7	Intact	scaffold116	14023651	14024523	+
	American_Crow_Tas2r1	Intact	scaffold44	3712104	3711139	-
	American_Crow_Tas2r9_Ps	Pseudogene	scaffold176	231755	232486	+
	American_Crow_Tas2r8_P	Partial	scaffold176	235228/246101	236057/246932	+
	American_Crow_Tas2r2	Intact	scaffold176	259591	260457	+
	American_Crow_Tas2r10_Ps	Pseudogene	scaffold176	261052	261675	+
Zebra Finch	Zebra_Finch_Tas2r7	Intact	197886634	34532	33597	-
	Zebra_Finch_Tas2r1	Intact	197886634	31495	30563	-
	Zebra_Finch_Tas2r3	Intact	197886632	4989	4054	-
	Zebra_Finch_Tas2r5	Intact	197889227	1229	2182	+
	Zebra_Finch_Tas2r4	Intact	197889227	5553	6506	+
	Zebra_Finch_Tas2r2	Intact	197889227	8615	9556	+
	Zebra_Finch_Tas2r6	Intact	197889228	25001	25951	+
	Zebra_Finch_Tas2r8_Ps	Pseudogene	197886466	66259	65558	-
Budgerigar	Budgerigar_Tas2r1	Intact	scf900160277026	5924866	5923916	-
	Budgerigar_Tas2r2_Ps	Pseudogene	scf900160277078	2394556	2394238	-
Kea	Kea_Tas2r1	Intact	scaffold708	17773	16823	-
	Kea_Tas2r2_Ps	Pseudogene	scaffold2803	24786	25289	+
Peregrine Falcon	Falco_peregrinus_Tas2r1	Intact	scaffold36_1	974909	973959	-
	Falco_peregrinus_Tas2r2	Intact	scaffold236_2	252192	251236	-
Red-legged Seriema	Red-legged_Seriema_Tas2r1	Intact	scaffold4031	30673	31608	+
	Red-legged_Seriema_Tas2r2	Intact	scaffold19391	18028	17084	-
Carmine Bee-eater	Carmine_Bee-eater_Tas2r1	Intact	scaffold3372	87990	88961	+

	Carmine_Bee-eater_Tas2r2_P	Partial	scaffold19532	33317	33655	+
	Carmine_Bee-eater_Tas2r3_P	Partial	scaffold20440	408	1	-
	Carmine_Bee-eater_Tas2r4_P	Partial	scaffold41834	665	153	-
Downy Woodpecker	Downy_Woodpecker_Tas2r3	Intact	scaffold415	197347	198282	+
	Downy_Woodpecker_Tas2r1	Intact	scaffold167	1583542	1582640	-
	Downy_Woodpecker_Tas2r5_Ps	Pseudogene	scaffold167	1203820	1202920	-
	Downy_Woodpecker_Tas2r2	Intact	scaffold167	1199105	1198152	-
	Downy_Woodpecker_Tas2r4	Intact	scaffold167	1194675	1193722	-
Rhinoceros Hornbill	Rhinoceros_Hornbill_Tas2r1	Intact	scaffold12344	27568	26621	-
	Rhinoceros_Hornbill_Tas2r2_P	Partial	scaffold13313	15130	14588	-
	Rhinoceros_Hornbill_Tas2r3_Ps	Pseudogene	scaffold16794	48703	49158	+
Bar-tailed Trogon	Bar-tailed_Trogon_Tas2r1	Intact	C12131352	2119	1166	-
	Bar-tailed_Trogon_Tas2r2	Intact	scaffold21851	7714	6761	-
	Bar-tailed_Trogon_Tas2r3	Intact	scaffold31381	3617	2655	-
	Bar-tailed_Trogon_Tas2r4	Intact	scaffold1004	9023	8070	-
	Bar-tailed_Trogon_Tas2r5	Intact	scaffold11293	1524	586	-
	Bar-tailed_Trogon_Tas2r6_P	Partial	scaffold6731	959	179	-
	Bar-tailed_Trogon_Tas2r7_P	Partial	scaffold2738	3688	3284	-
	Bar-tailed_Trogon_Tas2r8_P	Partial	scaffold23126	870	280	-
	Bar-tailed_Trogon_Tas2r9_P	Partial	scaffold15085	4	248	+
	Bar-tailed_Trogon_Tas2r10_P	Partial	scaffold22121	895	1149	+
	Bar-tailed_Trogon_Tas2r11_P	Partial	scaffold41964	431	3	-
	Bar-tailed_Trogon_Tas2r12_Ps	Pseudogene	scaffold21145	631	212	-
	Bar-tailed_Trogon_Tas2r13_Ps	Pseudogene	scaffold5893	3341	3673	+
Cuckoo Roller	Cuckoo_Roller_Tas2r1	Intact	scaffold14764	1156	2103	+
	Cuckoo_Roller_Tas2r2	Intact	scaffold9319	79935	80882	+
Speckled Mousebird	Speckled_Mousebird_Tas2r1	Intact	scaffold12255	22430	21483	-
	Speckled_Mousebird_Tas2r2_P	Partial	scaffold37637	864	1	-
	Speckled_Mousebird_Tas2r3_P	Partial	scaffold38140	110	627	+
	Speckled_Mousebird_Tas2r4_P	Partial	scaffold37670	327	1	-

Barn Owl	Barn_Owl_Tas2r1	Intact	scaffold2332	27837	26890	-
	Barn_Owl_Tas2r2	Intact	scaffold45413	3568	2615	-
White-tailed Eagle	White-tailed_Eagle_Tas2r1	Intact	scaffold37208	12737	13684	+
	White-tailed_Eagle_Tas2r2	Intact	scaffold28067	1469	2404	+
	White-tailed_Eagle_Tas2r3_Ps	Pseudogene	scaffold6740	109178	109689	+
Bald Eagle	Bald_Eagle_Tas2r1	Intact	Scaffold417	20795	21742	+
	Bald_Eagle_Tas2r2	Intact	Scaffold1158	116576	117511	+
	Bald_Eagle_Tas2r3_Ps	Pseudogene	Scaffold2485	264533	264132	-
Turkey Vulture	Turkey_Vulture_Tas2r1	Intact	scaffold669	17751	16822	-
	Turkey_Vulture_Tas2r2	Intact	scaffold12515	10755	9820	-
	Turkey_Vulture_Tas2r3_Ps	Pseudogene	scaffold8688	22181	21309	-
Dalmatian Pelican	Dalmatian_Pelican_Tas2r1	Intact	scaffold5020	54960	55913	+
	Dalmatian_Pelican_Tas2r2_Ps	Pseudogene	scaffold1017	134426	133610	-
Little Egret	Little_Egret_Tas2r2	Intact	scaffold323	734669	735129	+
	Little_Egret_Tas2r1	Intact	scaffold235	857838	858791	+
	Little_Egret_Tas2r3_Ps	Pseudogene	scaffold85	4378460	4377559	-
Crested Ibis	Crested_Ibis_Tas2r1	Intact	Scaffold6	7962604	7963572	+
	Crested_Ibis_Tas2r2	Intact	Scaffold155	4229721	4230674	+
Great Cormorant	Great_Cormorant_Tas2r1	Intact	scaffold16155	44227	45180	+
	Great_Cormorant_Tas2r2_Ps	Pseudogene	scaffold1307	8247	7459	-
Northern Fulmar	Northern_Fulmar_Tas2r1	Intact	scaffold3220	32172	33119	+
	Northern_Fulmar_Tas2r2	Intact	scaffold2043	1921	968	-
Emperor Penguin	Emperor_Penguin_Tas2r1_Ps	Pseudogene	Scaffold405	350048	350730	+
	Emperor_Penguin_Tas2r2_Ps	Pseudogene	Scaffold231	4670477	4669656	-
	Emperor_Penguin_Tas2r3_Ps	Pseudogene	Scaffold57	3998393	3997770	-
Adelie Penguin	Adelie_Penguin_Tas2r1_Ps	Pseudogene	Scaffold84	2982957	2983310	+
	Adelie_Penguin_Tas2r2_Ps	Pseudogene	Scaffold120	373785	374467	+
	Adelie_Penguin_Tas2r3_Ps	Pseudogene	Scaffold121	4214242	4215192	+
Red-throated Loon	Red-throated_Loon_Tas2r1_Ps	Pseudogene	scaffold5827	40213	40977	+
	Red-throated_Loon_Tas2r2_Ps	Pseudogene	scaffold9999	6330	7052	+

	Red-throated_Loon_Tas2r3_Ps	Pseudogene	scaffold6843	3746	4108	+
White-tailed Tropicbird	White-tailed_Tropicbird_Tas2r1	Intact	scaffold4143	36540	37490	+
	White-tailed_Tropicbird_Tas2r2_Ps	Pseudogene	scaffold8246	28218	28808	+
Sunbittern	Sunbittern_Tas2r1	Intact	scaffold37432	10666	11613	+
	Sunbittern_Tas2r2	Intact	C15859869	1291	338	-
Killdeer	Killdeer_Tas2r1	Intact	scaffold1	8183821	8184768	+
	Killdeer_Tas2r2	Intact	scaffold301	300207	301139	+
	Killdeer_Tas2r3	Intact	scaffold800	300112	301065	+
Grey Crowned Crane	Grey_Crowned_Crane_Tas2r1	Intact	scaffold1927	37295	38248	+
	Grey_Crowned_Crane_Tas2r2	Intact	scaffold12917	15309	14374	-
	Grey_Crowned_Crane_Tas2r3_Ps	Pseudogene	scaffold8931	32614	32237	-
Hoatzin	Hoatzin_Tas2r1	Intact	scaffold1028	1673	720	-
	Hoatzin_Tas2r2	Intact	scaffold659	3004	3957	+
	Hoatzin_Tas2r3	Intact	scaffold813	102136	103071	+
	Hoatzin_Tas2r4	Intact	scaffold256	1620801	1621754	+
Anna’s Hummingbird	Hummingbird_Tas2r3	Intact	scaffold127	2659677	2658724	-
	Hummingbird_Tas2r6	Intact	scaffold127	2655667	2654714	-
	Hummingbird_Tas2r4	Intact	scaffold33	3789761	3788835	-
	Hummingbird_Tas2r2	Intact	scaffold372	10650	9697	-
	Hummingbird_Tas2r12_Ps	Pseudogene	scaffold372	7772	7377	-
	Hummingbird_Tas2r5	Intact	scaffold372	5703	4750	-
	Hummingbird_Tas2r1	Intact	scaffold372	1304	342	-
	Hummingbird_Tas2r7_P	Partial	C10465731	3	224	+
	Hummingbird_Tas2r8_P	Partial	C10690480	3	242	+
	Hummingbird_Tas2r9_P	Partial	C10499692	242	3	-
	Hummingbird_Tas2r10_P	Partial	C10429533	3	203	+
	Hummingbird_Tas2r11_Ps	Pseudogene	C10902572	676	162	-
Chimney Swift	Chimney_Swift_Tas2r1	Intact	scaffold46	6393863	6394828	+
	Chimney_Swift_Tas2r4	Intact	scaffold277	283732	282785	-
	Chimney_Swift_Tas2r2	Intact	scaffold99	2644851	2643886	-

	Chimney_Swift_Tas2r3	Intact	scaffold99	2641505	2640543	-
	Chimney_Swift_Tas2r6_P	Partial	scaffold99	2636740	2636288	-
	Chimney_Swift_Tas2r5_P	Partial	scaffold46	6389989	6390327	+
Chuck-will's-widow	Chuck-wills-widow_Tas2r1	Intact	scaffold396	2121	3047	+
	Chuck-wills-widow_Tas2r2	Intact	scaffold1914	4972	4025	-
	Chuck-wills-widow_Tas2r3	Intact	scaffold1000	93270	92323	-
MacQueen's Bustard	MacQueens_Bustard_Tas2r1	Intact	scaffold23313	3506	2580	-
	MacQueens_Bustard_Tas2r2	Intact	scaffold414	82836	81925	-
	MacQueens_Bustard_Tas2r3_P	Partial	C15284394	1	474	+
	MacQueens_Bustard_Tas2r4_P	Partial	C15304738	265	525	+
Red-crested Turaco	Red-crested_Turaco_Tas2r1	Intact	scaffold2314	23361	22441	-
	Red-crested_Turaco_Tas2r2	Intact	scaffold34572	20881	19928	-
	Red-crested_Turaco_Tas2r3	Intact	scaffold18533	42888	41941	-
Common Cuckoo	Common_Cuckoo_Tas2r1	Intact	scaffold15	9317171	9316224	-
	Common_Cuckoo_Tas2r2	Intact	scaffold463	861923	860972	-
	Common_Cuckoo_Tas2r3_P	Partial	scaffold463	832513	832188	-
Brown Mesite	Brown_Mesite_Tas2r1	Intact	scaffold7920	19231	18269	-
	Brown_Mesite_Tas2r2	Intact	scaffold2045	26762	27703	+
	Brown_Mesite_Tas2r3	Intact	scaffold16819	19640	20575	+
Yellow-throated Sandgrouse	Yellow-throated_Sandgrouse_Tas2r1	Intact	scaffold1013	45299	46249	+
	Yellow-throated_Sandgrouse_Tas2r2_Ps	Pseudogene	scaffold28979	19334	19654	+
	Yellow-throated_Sandgrouse_Tas2r3_Ps	Pseudogene	scaffold15952	19934	19538	-
Domestic Pigeon	Domestic_Pigeon_Tas2r1	Intact	scaffold589	321699	322652	+
American Flamingo	American_Flamingo_Tas2r1	Intact	scaffold3389	6195	7154	+
	American_Flamingo_Tas2r2	Intact	scaffold9414	12146	11193	-
Great Crested Grebe	Great_Crested_Grebe_Tas2r1	Intact	scaffold16170	8914	7964	-
	Great_Crested_Grebe_Tas2r2_Ps	Pseudogene	scaffold2598	19885	20763	+
Turkey	Turkey_Tas2r1	Intact	chromosome2	115831982	115830963	-
	Turkey_Tas2r2	Intact	chromosome1	199170357	199171292	+
	Turkey_Tas2r3_Ps	Pseudogene	chromosome1	81484150	81483545	-

	Turkey_Tas2r4_Ps	Pseudogene	chromosome1	81511020	81510565	-
Chicken	Chicken_Tas2r1	Intact	354539478	114300	115265	+
	Chicken_Tas2r2	Intact	354542216	41217	40282	-
	Chicken_Tas2r3	Intact	354541529	5258	6193	+
Peking Duck	Peking_Duck_Tas2r1	Intact	scaffold1150	546595	547530	+
	Peking_Duck_Tas2r2	Intact	scaffold470	320497	319553	-
	Peking_Duck_Tas2r3_P	Partial	C18799681	326	3	-
	Peking_Duck_Tas2r4_P	Partial	scaffold2869	1	729	+
White-throated Tinamou	White-throated_Tinamou_Tas2r1	Intact	scaffold1945	19999	19064	-
	White-throated_Tinamou_Tas2r2	Intact	scaffold252	624516	625445	+
Common Ostrich	Common_Ostrich_Tas2r1	Intact	scaffold494	1849648	1848713	-
	Common_Ostrich_Tas2r2_Ps	Pseudogene	scaffold966	256186	256888	+

Table S6. Tandem duplicated *Tas2r* genes and the lengths of their scaffolds.

Species	Scaffold	Scaffold length (bp)	*Tas2r* gene number
**Rifleman**	scaffold4455	79059	2
	scaffold12242	12473	2
**Golden-collared Manakin**	scaffold114	2564601	5
**Medium Ground Finch**	scaffold52	279058	3
	scaffold1047	566768	3
**American Crow**	scaffold116	16488145	5
	scaffold176	604260	4
**Downy Woodpecker**	scaffold167	3849947	4
**Anna's Hummingbird**	scaffold127	2661235	2
	scaffold372	3831364	4
**Chimney Swift**	scaffold99	2651650	3
**Zebra Finch**	197886634	36224	2
	197889227	112379	3

Table S7. Potential gene conversion events between avian *Tas2r* genes.

Sequence pair	*P* value	Fragment length
Zebra_Finch_*Tas2r1* and Zebra_Finch_*Tas2r3*	0.01047	71 aa
Zebra_Finch_*Tas2r3* and Zebra_Finch_*Tas2r7*	0.02702	75 aa

Figure Legends

Figure S1. The Bayesian Inference (BI) phylogenetic tree with detailed species and gene names, and Bayesian posterior probabilities as percentages that were not shown in figure 1.

Figure S2. The Bayesian phylogenetic tree topology, where nodes with Bayesian posterior probabilities below 50% were collapsed. See details in figure S1.

Figure S3. The neighbor-joining phylogenetic tree with pairwise-deletion of gaps.

Figure S4. The neighbor-joining phylogenetic tree with complete-deletion of gaps.

Figure S5. While coded the red-legged seriema as 1, the repeated phylogenetically independent contrast (PIC) analysis confirmed the correlation between diet codes and putatively functional *Tas2r* gene numbers (A), and revealed a same trend between PICs of diet codes and those of total *Tas2r* gene numbers (B).

Figure S6. No correlation between the contig N50 length of each genome and the fraction of partial *Tas2r* genes.

Figure S1

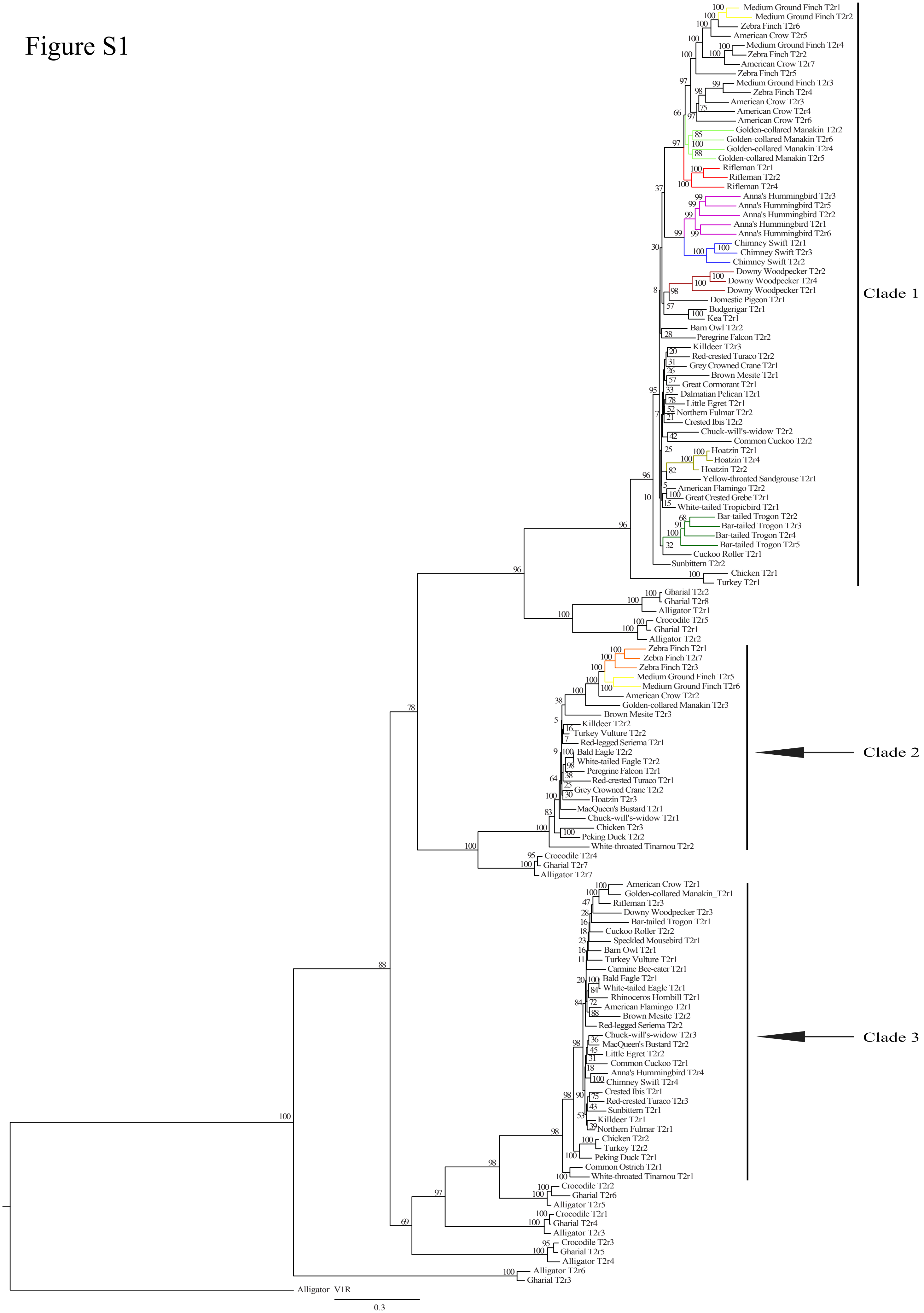
Medium Ground Finch T2r1
Medium Ground Finch T2r2
Zebra Finch T2r6
American Crow T2r5
Medium Ground Finch T2r4
Zebra Finch T2r2
American Crow T2r7
Zebra Finch T2r5
Medium Ground Finch T2r3
Zebra Finch T2r4
American Crow T2r3
American Crow T2r4
American Crow T2r6
Golden-collared Manakin T2r2
Golden-collared Manakin T2r6
Golden-collared Manakin T2r4
Golden-collared Manakin T2r5
Rifleman T2r1
Rifleman T2r2
Rifleman T2r4
Anna's Hummingbird T2r3
Anna's Hummingbird T2r5
Anna's Hummingbird T2r2
Anna's Hummingbird T2r1
Anna's Hummingbird T2r6
Chimney Swift T2r1
Chimney Swift T2r3
Chimney Swift T2r2
Downy Woodpecker T2r2
Downy Woodpecker T2r4
Downy Woodpecker T2r1
Domestic Pigeon T2r1
Budgerigar T2r1
Kea T2r1
Barn Owl T2r2
Peregrine Falcon T2r2
Killdeer T2r3
Red-crested Turaco T2r2
Grey Crowned Crane T2r1
Brown Mesite T2r1
Great Cormorant T2r1
Dalmatian Pelican T2r1
Little Egret T2r1
Northern Fulmar T2r2
Crested Ibis T2r2
Chuck-will's-widow T2r2
Common Cuckoo T2r2
Hoatzin T2r1
Hoatzin T2r4
Hoatzin T2r2
Yellow-throated Sandgrouse T2r1
American Flamingo T2r2
Great Crested Grebe T2r1
White-tailed Tropicbird T2r1
Bar-tailed Trogon T2r2
Bar-tailed Trogon T2r3
Bar-tailed Trogon T2r4
Bar-tailed Trogon T2r5
Cuckoo Roller T2r1
Sunbittern T2r2
Chicken T2r1
Turkey T2r1
Clade 1
Gharial T2r2
Gharial T2r8
Alligator T2r1
Crocodile T2r5
Gharial T2r1
Alligator T2r2
Zebra Finch T2r1
Zebra Finch T2r7
Zebra Finch T2r3
Medium Ground Finch T2r5
Medium Ground Finch T2r6
American Crow T2r2
Golden-collared Manakin T2r3
Brown Mesite T2r3
Killdeer T2r2
Turkey Vulture T2r2
Red-legged Seriema T2r1
Bald Eagle T2r2
White-tailed Eagle T2r2
Peregrine Falcon T2r1
Red-crested Turaco T2r1
Grey Crowned Crane T2r2
Hoatzin T2r3
MacQueen's Bustard T2r1
Chuck-will's-widow T2r1
Chicken T2r3
Peking Duck T2r2
White-throated Tinamou T2r2
Clade 2
Crocodile T2r4
Gharial T2r7
Alligator T2r7
American Crow T2r1
Golden-collared Manakin_T2r1
Rifleman T2r3
Downy Woodpecker T2r3
Bar-tailed Trogon T2r1
Cuckoo Roller T2r2
Speckled Mousebird T2r1
Barn Owl T2r1
Turkey Vulture T2r1
Carmine Bee-eater T2r1
Bald Eagle T2r1
White-tailed Eagle T2r1
Rhinoceros Hornbill T2r1
American Flamingo T2r1
Brown Mesite T2r2
Red-legged Seriema T2r2
Chuck-will's-widow T2r3
MacQueen's Bustard T2r2
Little Egret T2r2
Common Cuckoo T2r1
Anna's Hummingbird T2r4
Chimney Swift T2r4
Crested Ibis T2r1
Red-crested Turaco T2r3
Sunbittern T2r1
Killdeer T2r1
Northern Fulmar T2r1
Chicken T2r2
Turkey T2r2
Peking Duck T2r1
Common Ostrich T2r1
White-throated Tinamou T2r1
Clade 3
Crocodile T2r2
Gharial T2r6
Alligator T2r5
Crocodile T2r1
Gharial T2r4
Alligator T2r3
Crocodile T2r3
Gharial T2r5
Alligator T2r4
Alligator T2r6
Gharial T2r3
Alligator V1R
0.3

Figure S2

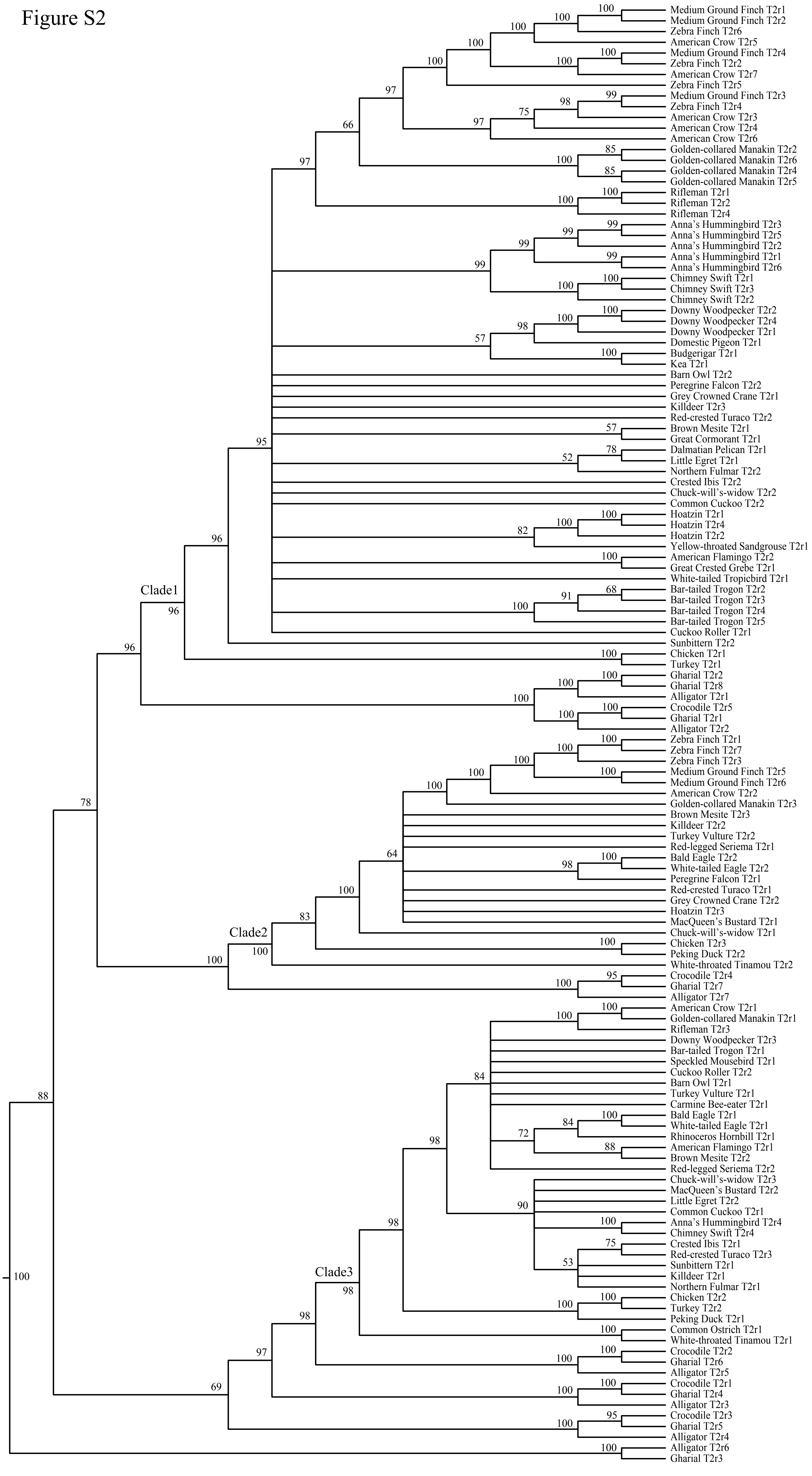
Medium Ground Finch T2r1
Medium Ground Finch T2r2
Zebra Finch T2r6
American Crow T2r5
Medium Ground Finch T2r4
Zebra Finch T2r2
American Crow T2r7
Zebra Finch T2r5
Medium Ground Finch T2r3
Zebra Finch T2r4
American Crow T2r3
American Crow T2r4
American Crow T2r6
Golden-collared Manakin T2r2
Golden-collared Manakin T2r6
Golden-collared Manakin T2r4
Golden-collared Manakin T2r5
Rifleman T2r1
Rifleman T2r2
Rifleman T2r4
Anna's Hummingbird T2r3
Anna's Hummingbird T2r5
Anna's Hummingbird T2r2
Anna's Hummingbird T2r1
Anna's Hummingbird T2r6
Chimney Swift T2r1
Chimney Swift T2r3
Chimney Swift T2r2
Downy Woodpecker T2r2
Downy Woodpecker T2r4
Downy Woodpecker T2r1
Domestic Pigeon T2r1
Budgerigar T2r1
Kea T2r1
Barn Owl T2r2
Peregrine Falcon T2r2
Grey Crowned Crane T2r1
Killdeer T2r3
Red-crested Turaco T2r2
Brown Mesite T2r1
Great Cormorant T2r1
Dalmatian Pelican T2r1
Little Egret T2r1
Northern Fulmar T2r2
Crested Ibis T2r2
Chuck-will's-widow T2r2
Common Cuckoo T2r2
Hoatzin T2r1
Hoatzin T2r4
Hoatzin T2r2
Yellow-throated Sandgrouse T2r1
American Flamingo T2r2
Great Crested Grebe T2r1
White-tailed Tropicbird T2r1
Bar-tailed Trogon T2r2
Bar-tailed Trogon T2r3
Bar-tailed Trogon T2r4
Bar-tailed Trogon T2r5
Cuckoo Roller T2r1
Sunbittern T2r2
Chicken T2r1
Turkey T2r1
Gharial T2r2
Gharial T2r8
Alligator T2r1
Crocodile T2r5
Gharial T2r1
Alligator T2r2
Zebra Finch T2r1
Zebra Finch T2r7
Zebra Finch T2r3
Medium Ground Finch T2r5
Medium Ground Finch T2r6
American Crow T2r2
Golden-collared Manakin T2r3
Brown Mesite T2r3
Killdeer T2r2
Turkey Vulture T2r2
Red-legged Seriema T2r1
Bald Eagle T2r2
White-tailed Eagle T2r2
Peregrine Falcon T2r1
Red-crested Turaco T2r1
Grey Crowned Crane T2r2
Hoatzin T2r3
MacQueen's Bustard T2r1
Chuck-will's-widow T2r1
Chicken T2r3
Peking Duck T2r2
White-throated Tinamou T2r2
Crocodile T2r4
Gharial T2r7
Alligator T2r7
American Crow T2r1
Golden-collared Manakin T2r1
Rifleman T2r3
Downy Woodpecker T2r3
Bar-tailed Trogon T2r1
Speckled Mousebird T2r1
Cuckoo Roller T2r2
Barn Owl T2r1
Turkey Vulture T2r1
Carmine Bee-eater T2r1
Bald Eagle T2r1
White-tailed Eagle T2r1
Rhinoceros Hornbill T2r1
American Flamingo T2r1
Brown Mesite T2r2
Red-legged Seriema T2r2
Chuck-will's-widow T2r3
MacQueen's Bustard T2r2
Little Egret T2r2
Common Cuckoo T2r1
Anna's Hummingbird T2r4
Chimney Swift T2r4
Crested Ibis T2r1
Red-crested Turaco T2r3
Sunbittern T2r1
Killdeer T2r1
Northern Fulmar T2r1
Chicken T2r2
Turkey T2r2
Peking Duck T2r1
Common Ostrich T2r1
White-throated Tinamou T2r1
Crocodile T2r2
Gharial T2r6
Alligator T2r5
Crocodile T2r1
Gharial T2r4
Alligator T2r3
Crocodile T2r3
Gharial T2r5
Alligator T2r4
Alligator T2r6
Gharial T2r3
Clade1
Clade2
Clade3

Figure S3

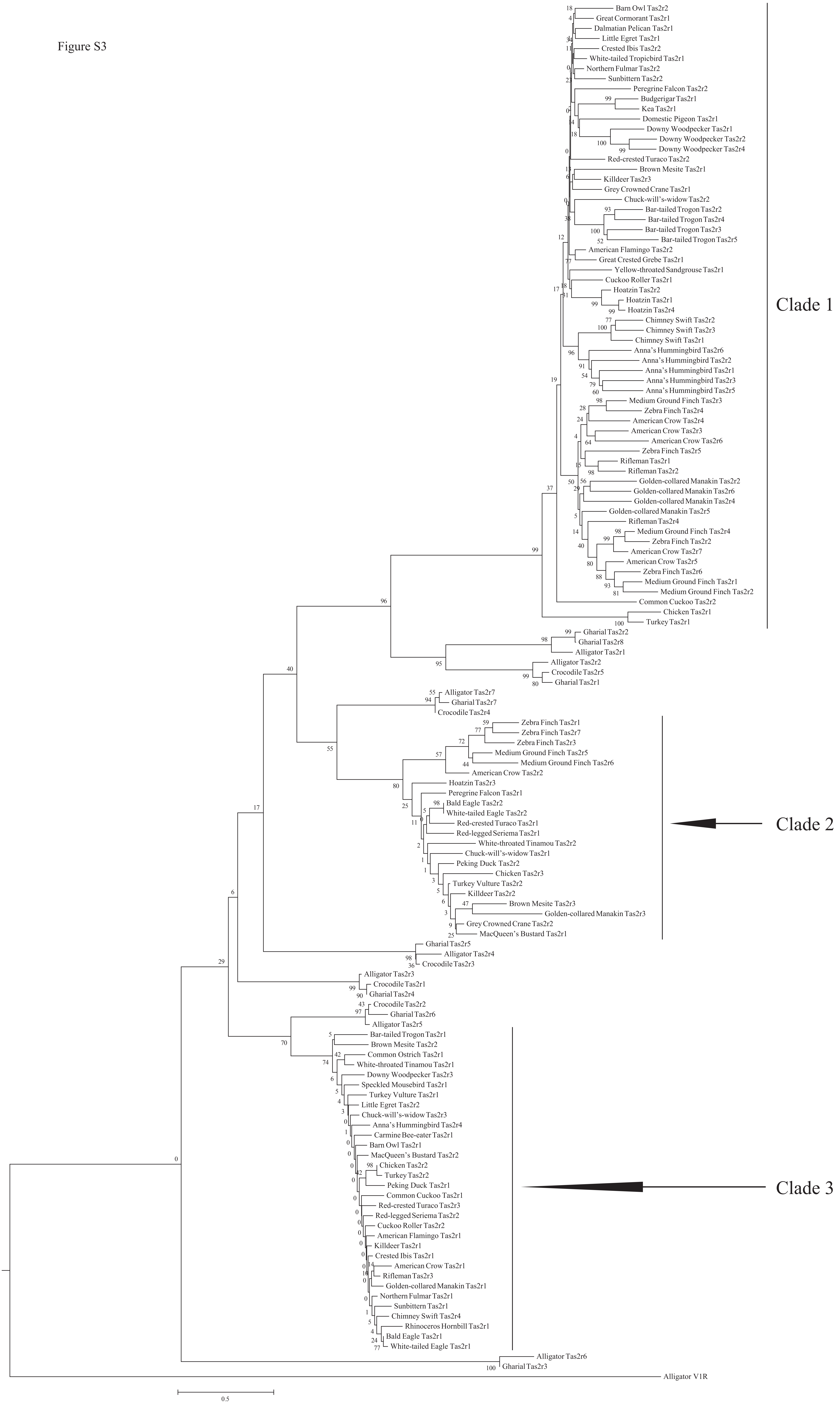

Barn Owl Tas2r2
Great Cormorant Tas2r1
Dalmatian Pelican Tas2r1
Little Egret Tas2r1
Crested Ibis Tas2r2
White-tailed Tropicbird Tas2r1
Northern Fulmar Tas2r2
Sunbittern Tas2r2
Peregrine Falcon Tas2r2
Budgerigar Tas2r1
Kea Tas2r1
Domestic Pigeon Tas2r1
Downy Woodpecker Tas2r1
Downy Woodpecker Tas2r2
Downy Woodpecker Tas2r4
Red-crested Turaco Tas2r2
Brown Mesite Tas2r1
Killdeer Tas2r3
Grey Crowned Crane Tas2r1
Chuck-will's-widow Tas2r2
Bar-tailed Trogon Tas2r2
Bar-tailed Trogon Tas2r4
Bar-tailed Trogon Tas2r3
Bar-tailed Trogon Tas2r5
American Flamingo Tas2r2
Great Crested Grebe Tas2r1
Yellow-throated Sandgrouse Tas2r1
Cuckoo Roller Tas2r1
Hoatzin Tas2r2
Hoatzin Tas2r1
Hoatzin Tas2r4
Chimney Swift Tas2r2
Chimney Swift Tas2r3
Chimney Swift Tas2r1
Anna's Hummingbird Tas2r6
Anna's Hummingbird Tas2r2
Anna's Hummingbird Tas2r1
Anna's Hummingbird Tas2r3
Anna's Hummingbird Tas2r5
Medium Ground Finch Tas2r3
Zebra Finch Tas2r4
American Crow Tas2r4
American Crow Tas2r3
American Crow Tas2r6
Zebra Finch Tas2r5
Rifleman Tas2r1
Rifleman Tas2r2
Golden-collared Manakin Tas2r2
Golden-collared Manakin Tas2r6
Golden-collared Manakin Tas2r4
Golden-collared Manakin Tas2r5
Rifleman Tas2r4
Medium Ground Finch Tas2r4
Zebra Finch Tas2r2
American Crow Tas2r7
American Crow Tas2r5
Zebra Finch Tas2r6
Medium Ground Finch Tas2r1
Medium Ground Finch Tas2r2
Common Cuckoo Tas2r2
Chicken Tas2r1
Turkey Tas2r1
Clade 1
Gharial Tas2r2
Gharial Tas2r8
Alligator Tas2r1
Alligator Tas2r2
Crocodile Tas2r5
Gharial Tas2r1
Alligator Tas2r7
Gharial Tas2r7
Crocodile Tas2r4
Zebra Finch Tas2r1
Zebra Finch Tas2r7
Zebra Finch Tas2r3
Medium Ground Finch Tas2r5
Medium Ground Finch Tas2r6
American Crow Tas2r2
Hoatzin Tas2r3
Peregrine Falcon Tas2r1
Bald Eagle Tas2r2
White-tailed Eagle Tas2r2
Red-crested Turaco Tas2r1
Red-legged Seriema Tas2r1
White-throated Tinamou Tas2r2
Chuck-will's-widow Tas2r1
Peking Duck Tas2r2
Chicken Tas2r3
Turkey Vulture Tas2r2
Killdeer Tas2r2
Brown Mesite Tas2r3
Golden-collared Manakin Tas2r3
Grey Crowned Crane Tas2r2
MacQueen's Bustard Tas2r1
Clade 2
Gharial Tas2r5
Alligator Tas2r4
Crocodile Tas2r3
Alligator Tas2r3
Crocodile Tas2r1
Gharial Tas2r4
Crocodile Tas2r2
Gharial Tas2r6
Alligator Tas2r5
Bar-tailed Trogon Tas2r1
Brown Mesite Tas2r2
Common Ostrich Tas2r1
White-throated Tinamou Tas2r1
Downy Woodpecker Tas2r3
Speckled Mousebird Tas2r1
Turkey Vulture Tas2r1
Little Egret Tas2r2
Chuck-will's-widow Tas2r3
Anna's Hummingbird Tas2r4
Carmine Bee-eater Tas2r1
Barn Owl Tas2r1
MacQueen's Bustard Tas2r2
Chicken Tas2r2
Turkey Tas2r2
Peking Duck Tas2r1
Common Cuckoo Tas2r1
Red-crested Turaco Tas2r3
Red-legged Seriema Tas2r2
Cuckoo Roller Tas2r2
American Flamingo Tas2r1
Killdeer Tas2r1
Crested Ibis Tas2r1
American Crow Tas2r1
Rifleman Tas2r3
Golden-collared Manakin Tas2r1
Northern Fulmar Tas2r1
Sunbittern Tas2r1
Chimney Swift Tas2r4
Rhinoceros Hornbill Tas2r1
Bald Eagle Tas2r1
White-tailed Eagle Tas2r1
Clade 3
Alligator Tas2r6
Gharial Tas2r3
Alligator V1R
0.5

Figure S4

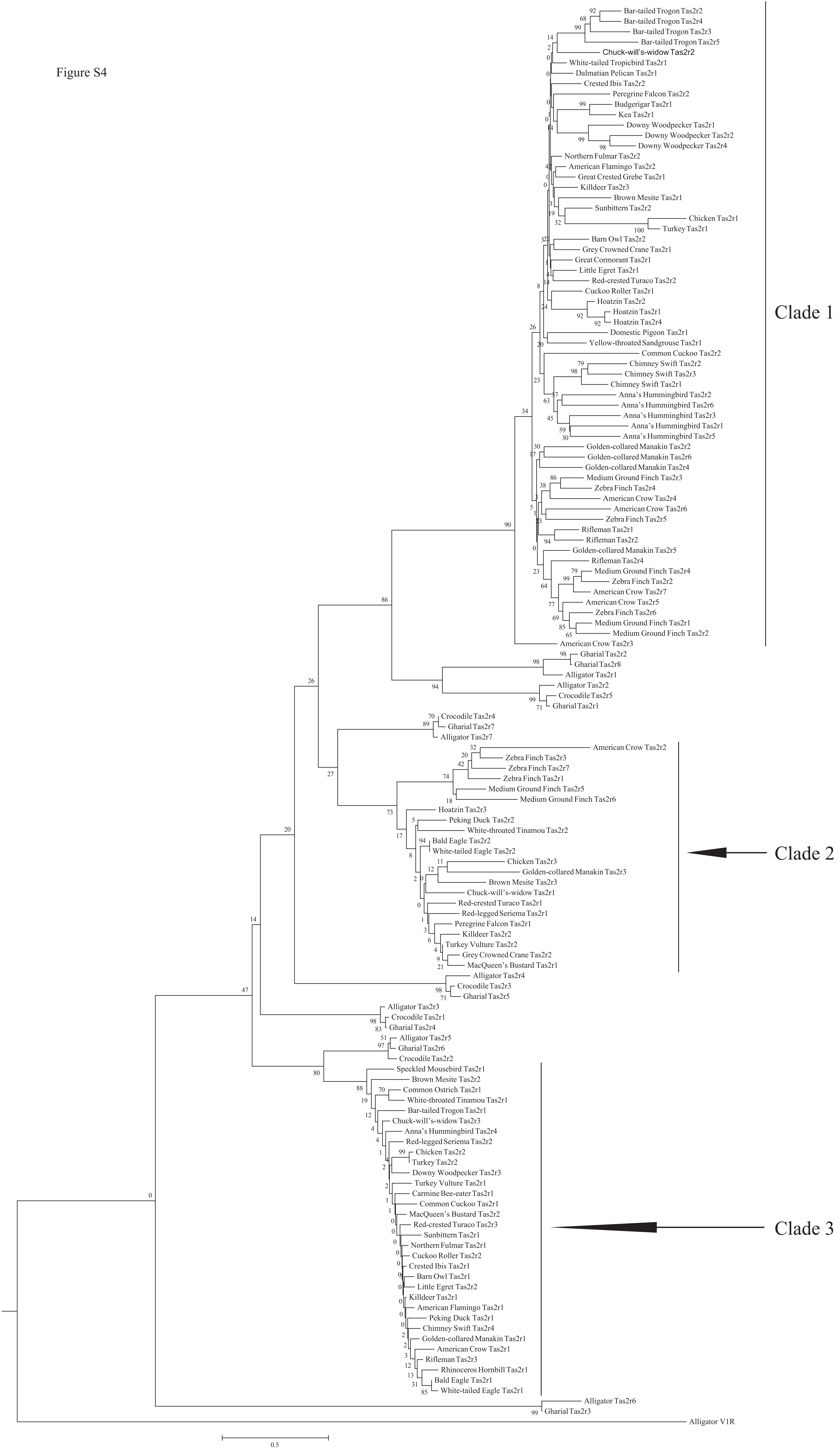
Bar-tailed Trogon Tas2r2
Bar-tailed Trogon Tas2r4
Bar-tailed Trogon Tas2r3
Bar-tailed Trogon Tas2r5
Chuck-will's-widow Tas2r2
White-tailed Tropicbird Tas2r1
Dalmatian Pelican Tas2r1
Crested Ibis Tas2r2
Peregrine Falcon Tas2r2
Budgerigar Tas2r1
Kea Tas2r1
Downy Woodpecker Tas2r1
Downy Woodpecker Tas2r2
Downy Woodpecker Tas2r4
Northern Fulmar Tas2r2
American Flamingo Tas2r2
Great Crested Grebe Tas2r1
Killdeer Tas2r3
Brown Mesite Tas2r1
Sunbittern Tas2r2
Chicken Tas2r1
Turkey Tas2r1
Barn Owl Tas2r2
Grey Crowned Crane Tas2r1
Great Cormorant Tas2r1
Little Egret Tas2r1
Red-crested Turaco Tas2r2
Cuckoo Roller Tas2r1
Hoatzin Tas2r2
Hoatzin Tas2r1
Hoatzin Tas2r4
Domestic Pigeon Tas2r1
Yellow-throated Sandgrouse Tas2r1
Common Cuckoo Tas2r2
Chimney Swift Tas2r2
Chimney Swift Tas2r3
Chimney Swift Tas2r1
Anna's Hummingbird Tas2r2
Anna's Hummingbird Tas2r6
Anna's Hummingbird Tas2r3
Anna's Hummingbird Tas2r1
Anna's Hummingbird Tas2r5
Golden-collared Manakin Tas2r2
Golden-collared Manakin Tas2r6
Golden-collared Manakin Tas2r4
Medium Ground Finch Tas2r3
Zebra Finch Tas2r4
American Crow Tas2r4
American Crow Tas2r6
Zebra Finch Tas2r5
Rifleman Tas2r1
Rifleman Tas2r2
Golden-collared Manakin Tas2r5
Rifleman Tas2r4
Medium Ground Finch Tas2r4
Zebra Finch Tas2r2
American Crow Tas2r7
American Crow Tas2r5
Zebra Finch Tas2r6
Medium Ground Finch Tas2r1
Medium Ground Finch Tas2r2
American Crow Tas2r3
Clade 1
Gharial Tas2r2
Gharial Tas2r8
Alligator Tas2r1
Alligator Tas2r2
Crocodile Tas2r5
Gharial Tas2r1
Crocodile Tas2r4
Gharial Tas2r7
Alligator Tas2r7
American Crow Tas2r2
Zebra Finch Tas2r3
Zebra Finch Tas2r7
Zebra Finch Tas2r1
Medium Ground Finch Tas2r5
Medium Ground Finch Tas2r6
Hoatzin Tas2r3
Peking Duck Tas2r2
White-throated Tinamou Tas2r2
Bald Eagle Tas2r2
White-tailed Eagle Tas2r2
Chicken Tas2r3
Golden-collared Manakin Tas2r3
Brown Mesite Tas2r3
Chuck-will's-widow Tas2r1
Red-crested Turaco Tas2r1
Red-legged Seriema Tas2r1
Peregrine Falcon Tas2r1
Killdeer Tas2r2
Turkey Vulture Tas2r2
Grey Crowned Crane Tas2r2
MacQueen's Bustard Tas2r1
Clade 2
Alligator Tas2r4
Crocodile Tas2r3
Gharial Tas2r5
Alligator Tas2r3
Crocodile Tas2r1
Gharial Tas2r4
Alligator Tas2r5
Gharial Tas2r6
Crocodile Tas2r2
Speckled Mousebird Tas2r1
Brown Mesite Tas2r2
Common Ostrich Tas2r1
White-throated Tinamou Tas2r1
Bar-tailed Trogon Tas2r1
Chuck-will's-widow Tas2r3
Anna's Hummingbird Tas2r4
Red-legged Seriema Tas2r2
Chicken Tas2r2
Turkey Tas2r2
Downy Woodpecker Tas2r3
Turkey Vulture Tas2r1
Carmine Bee-eater Tas2r1
Common Cuckoo Tas2r1
MacQueen's Bustard Tas2r2
Red-crested Turaco Tas2r3
Sunbittern Tas2r1
Northern Fulmar Tas2r1
Cuckoo Roller Tas2r2
Crested Ibis Tas2r1
Barn Owl Tas2r1
Little Egret Tas2r2
Killdeer Tas2r1
American Flamingo Tas2r1
Peking Duck Tas2r1
Chimney Swift Tas2r4
Golden-collared Manakin Tas2r1
American Crow Tas2r1
Rifleman Tas2r3
Rhinoceros Hornbill Tas2r1
Bald Eagle Tas2r1
White-tailed Eagle Tas2r1
Clade 3
Alligator Tas2r6
Gharial Tas2r3
Alligator V1R
0.5

Figure S5

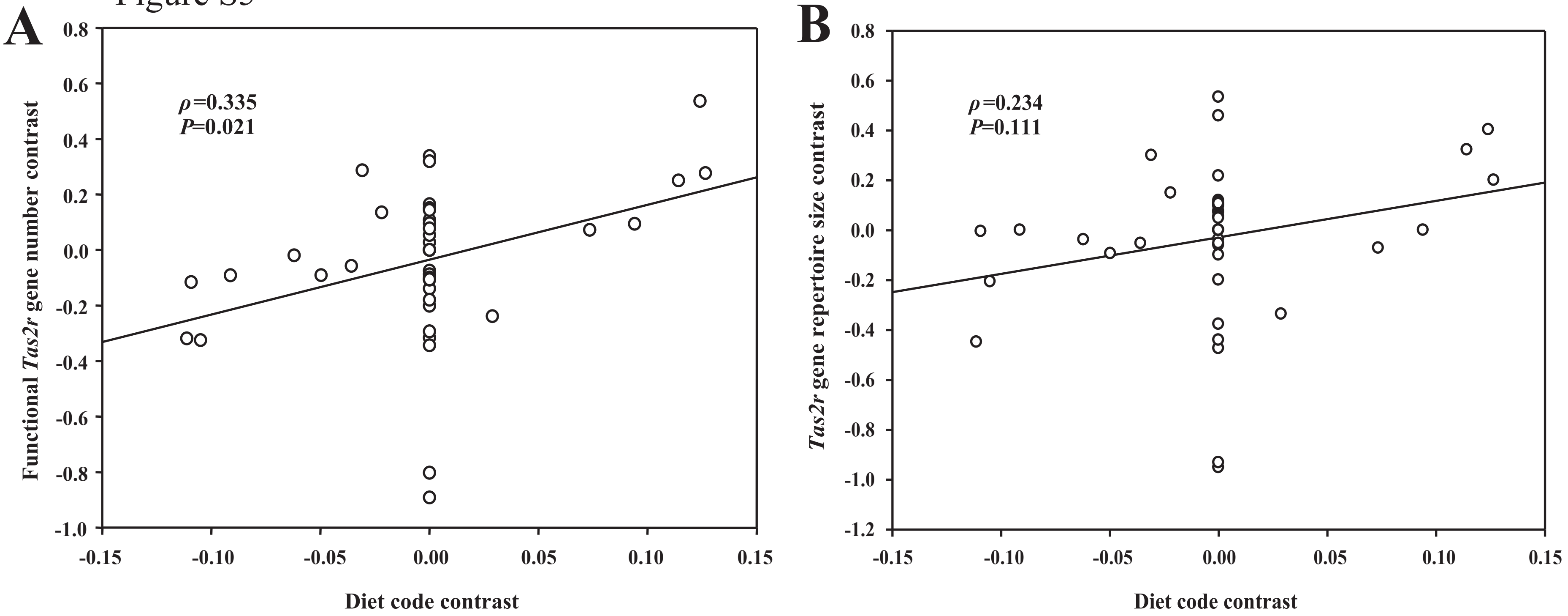

A
ρ=0.335
P=0.021
Functional Tas2r gene number contrast
Diet code contrast
0.8
0.6
0.4
0.2
0.0
-0.2
-0.4
-0.6
-0.8
-1.0
-0.15
-0.10
-0.05
0.00
0.05
0.10
0.15
B
ρ=0.234
P=0.111
Tas2r gene repertoire size contrast
Diet code contrast
0.8
0.6
0.4
0.2
0.0
-0.2
-0.4
-0.6
-0.8
-1.0
-1.2
-0.15
-0.10
-0.05
0.00
0.05
0.10
0.15

Figure S6

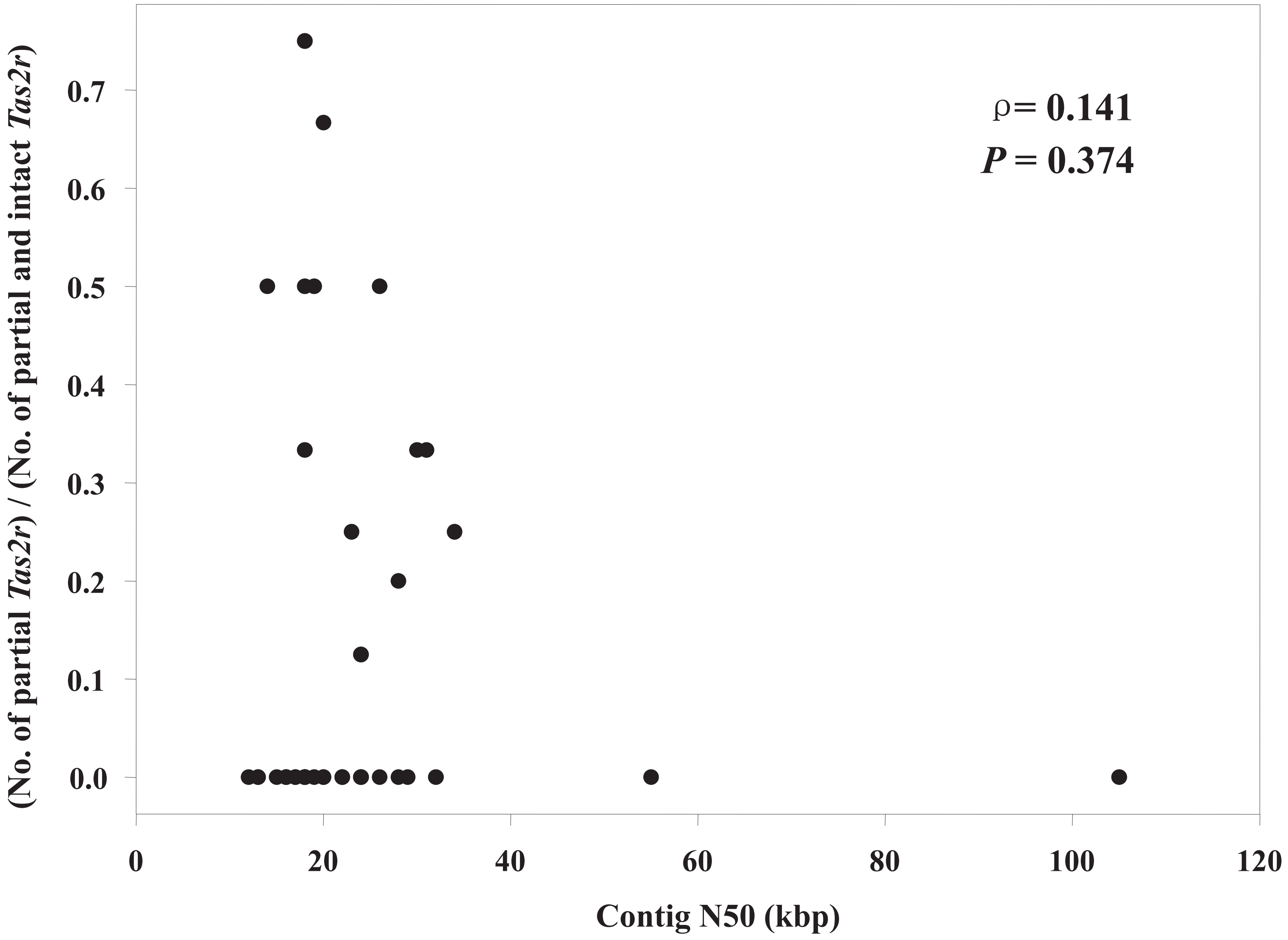

ρ= 0.141
P = 0.374
(No. of partial Tas2r) / (No. of partial and intact Tas2r)
0.0
0.1
0.2
0.3
0.4
0.5
0.6
0.7
0
20
40
60
80
100
120
Contig N50 (kbp)
